# Enhancing Morphology and Separation Performance of Polyamide 6,6 Membranes By Minimal Incorporation of Silver Decorated Graphene Oxide Nanoparticles

**DOI:** 10.1038/s41598-018-38060-x

**Published:** 2019-02-04

**Authors:** Ebrahim Mahmoudi, Law Yong Ng, Wei Lun Ang, Ying Tao Chung, Rosiah Rohani, Abdul Wahab Mohammad

**Affiliations:** 1 Chemical Engineering Programme, Faculty of Engineering and Built Environment, Universiti Kebangsaan Malaysia, 43600 Bangi, Selangor Darul Ehsan Malaysia; 2 Department of Chemical Engineering, Lee Kong Chian Faculty of Engineering and Science, Universiti Tunku Abdul Rahman, Jalan Sungai Long, Bandar Sungai Long, Cheras, 43000 Kajang, Selangor Darul Ehsan Malaysia; 3 Centre for Sustainable Process Technology (CESPRO), Faculty of Engineering and Built Environment, Universiti Kebangsaan Malaysia, 43600 Bangi, Selangor Darul Ehsan Malaysia

## Abstract

Nanomaterials can be incorporated in the synthesis of membrane to obtain mixed-matrix membrane with marked improvement in properties and performance. However, stability and dispersion of the nanomaterials in the membrane matrix, as well as the need to use high ratio of nanomaterials for obvious improvement of membrane properties, remain a major hurdle for commercialization. Hence, this study aims to investigate the improvement of polyamide 6,6 membrane properties with the incorporation of silver nanoparticles decorated on graphene oxide (Ag-GO) nanoplates and at the same time focus is given to the issues above. Graphene oxide nanoplates were synthesized using the modified Hummers’ method and decorated with silver before embedded into the polyamide 6,6 matrix. Physicochemical characterizations were conducted on both nanoplates and the mixed-matrix Ag-GO polyamide 6,6 membrane. The issues of Ag agglomeration and leaching were not observed, which could be attributed to the decoration of Ag on GO that helped to disperse the nanomaterials and provided a better anchor point for the attachment of Ag nanoparticles. The synthesized membrane showed marked improvement regarding flux (135% increment) and antifouling (40% lower irreversible fouling), which could be ascribed to the more negative charge of membrane surface (−14 ± 6 to −31 ± 3.8 mV) and hydrophilicity (46% enhancement) of the membranes. With minimal embedment of Ag nanoparticles, the membrane showed superior antibacterial property where the *E. coli* bacteria could not form a single colony on the membrane surface. Overall, the decoration of Ag on GO nanoplates could be a promising approach to resolve the agglomeration and leaching issues as well as reduce the amount of precious Ag in the synthesis of Ag-GO polyamide 6,6 membrane.

## Introduction

Water pollution and water shortage have become two of the most severe environmental problems globally. In order to curb these problems, advanced technologies such as membrane filtration process have been adopted to remove the pollutants from contaminated water and to produce clean water. Membrane technology has been successfully employed in various application, such as wastewater treatment, reclamation and drinking water production^1–3^. For instance, Kasim *et al*. showed that nanofiltration (NF) and ultrafiltration (UF) could remove heavy metal present in the groundwater, producing water safe for drinking consumption^4^. On the other hand, it was reported that membrane bioreactor (MBR) outperformed the conventional wastewater treatment process where it managed to treat the wastewater and convert it into high-quality reusable water. Not only MBR could remove pathogenic bacteria and viruses; it has a smaller footprint in comparison to conventional wastewater treatment that makes it an economically attractive treatment process^1,5,6^.

Though membrane technology has shown excellent performance in terms of water quality and removal of hazardous substances, its integrity in performance (especially permeation flux) has remained a major challenge for various applications^7^. Fouling is a major issue that will degrade the performance and lifespan of the membrane filtration unit. Various types of fouling such as organic fouling, inorganic fouling, and biofouling have been reported by the researchers as the most occurrence fouling issues encountered during the filtration process^8^. The deposition of foulants (e.g. natural organic matter, bacteria and scale precipitates) will block the permeation of water through the membrane, resulting in decreased permeation flux and rejection of hazardous substances^9^. For instance, the colloids and natural organic matter present in water were found to form a foulant cake layer on the membrane surface and reduce the permeation flux^10^. Mineral scales were reported to form on the membrane surface for desalination process, where the scales severely reduced the flux and salt rejection capability of the membrane system^11,12^. In addition, biofilm formation and microorganism growth on the membrane surface resulted in biofouling that not only reduced the water flux of MBR but also increased the transmembrane pressure and decreased the membrane lifespan, which literally could be translated to increment in operating cost^13–15^. Overall, fouling in any form affects the performance of the membrane process adversely by reducing its productivity (quality and quantity of filtered water) and the lifespan of the membrane module.

In order to minimize the impact of fouling, several approaches such as the design of membrane module^16^, operational parameters^17^ and membrane modification^18^ have been actively engaged by the researchers. Among these approaches, modification of the membrane materials and composition by incorporating nanomaterials to produce nanocomposite membrane appears to be a promising way to enhance the antifouling properties of the membrane (flux, hydrophilicity, and rejection)^19–21^. Numerous studies have shown that the combination of nanomaterials (Zinc oxide, Titanium dioxide, and silver nanoparticles) could enhance the membrane performance in permeability, selectivity, structure robustness, antifouling, antimicrobial and photodegradation properties^11^. However, there are several challenges associated with the synthesis of nanocomposite membrane. For instance, agglomeration of nanomaterials in the membrane matrix is a big issue where it would lead to undesirable changes in membrane properties, such as weakening membrane mechanical strength and lower water flux^22,23^. Leaching is another concern for the application of nanocomposite membrane^24^. It has been reported that nanomaterials have high tendency to leach out from the membrane matrix, rendering the enhancements of membrane properties futile^25^.

Another issue is the rationalization of using a high ratio of costly and valuable nanoparticles in the synthesis of nanocomposite membrane. Yi *et al*. reported that the blending of nanodiamonds into the Polyvinylidene fluoride (PVDF) membrane successfully increased the flux by 200% and Bovine serum albumin rejection by 20% higher. However, apart from the costly nanodiamonds, leaching is still an issue as these nanodiamonds did not have a proper anchor point to be strongly attached to the membrane structure^26^. Such a phenomenon was also observed by Zodrow *et al*. where silver nanoparticles were leached from the membrane after the filtration process. These leached nanoparticles could raise hazardous health risks for living organisms and end-users of the treated water, not to mention the associated cost of membrane replacement due to the loss of nanomaterials^27^. In a nutshell, the main challenges associated with the nanocomposite membrane are agglomeration, leaching, and rationalization of using a high ratio of costly nanomaterials.

To address this issue, a number of studies have recommended decorating the nanoparticles on graphene nanoplates before incorporating into the membrane matrix^28^. Literature has shown the potential use of graphene oxide (GO) as nanofillers to stabilize the nanoparticles in the membrane research^29^. For instance, silver nanoparticles could be decorated onto GO nanoplates to improve its dispersibility, preventing the agglomeration of Ag nanoparticles in the membrane matrix and subsequently enhancing the antimicrobial efficiency of the Ag as well^30,31^. At the same time, GO provided better anchor points for the nanomaterials, thus preventing it from leaching out from the polymer matrix^32^.

Polyamide (Nylon) 6,6 membrane has been widely used in water and wastewater treatment processes due to its great mechanical strength and hydrophilic nature^33^. Polyamide membrane shows stability in harsh physical and chemical conditions and this allows it to be used where other membranes are unsuitable or difficult to use. However, the study on the incorporation of Ag-decorated GO nanoplates into polyamide 6,6 membrane for improved properties has never been explored. Hence, this study aims to investigate the improvement of Polyamide 6,6 nanocomposite membrane properties (flux, rejection, and antimicrobial) with the incorporation of Ag-GO nanomaterials. The focus will be given to the three main issues associated with the synthesis of nanocomposite membrane: agglomeration, leaching, and quantity of nanomaterials.

## Experimental

### Materials

Extra pure and fine graphite with particle sizes ≤50 μm and formic acid (FA) (99%) were procured from Merck, Malaysia. Potassium permanganate (KMnO_4_), sulfuric acid (H_2_SO_4_) (98%), Poly(hexamethylene adipamide) (Polyamide 6,6) in pellet form and bovine serum albumin (BSA) (~66,000 Da) were obtained from Sigma-Aldrich, Malaysia. All the chemicals used were of analytical grade.

### Preparation and Characterization of Silver-decorated GO (Ag-GO)

Graphene oxide was synthesized by using the Hummers’ method from natural graphite powder as reported in the previous work^34^. Ag-GO nanoplates were then concocted by reducing the silver nitrate (AgNO_3_) with sodium borohydride (NaBH_4_) in aqueous GO solution (1 g/L)^30^. The Ag-GO nanoplates were washed with ultrapure water and freeze-dried before sent for characterization using the X-ray diffraction instrument (Bruker D8 Advance AXS X-ray diffractometer, United States). In addition, the transmission electron microscopy (TEM) was utilized to observe the Ag-GO nanocomposite (Philips CM200, model JEOLJEM 2100, Netherlands). The surface charge of nanomaterial was assessed by zeta potential measurement using Malvern Zeta Sizer Nano ZS (Malvern Instruments, UK) by applying field strength of 25 V/cm.

### Membrane Fabrication

A wet phase inversion method has been chosen for the fabrication of polyamide-Ag-GO blend membrane. The casting solution was formulated by dispersing the Ag-GO nanoplates in formic acid. The polymer solution was prepared with different weight ratios of polymer (polyamide 6,6) to solvent (formic acid), as summarized in Table 1. The nanoplates were ultra-sonicated for 30 minutes before mixing with the polymer solution. The mixture was heated (using silicon oil bath) at a constant temperature of 60 °C with the slow stirring rate of 300 rpm for 5 hr to prevent the formation of any air bubbles. The solution mixture was then ultra-sonicated for 30 minutes to ensure a better dispersion of the GO nanoplates in the mixture. During the fabrication process, a small amount of the casting solution was poured onto a clean glass plate and cast using Filmographe Doctor Blade 360099003 (Braive Instrument, Germany). The thickness of the membranes was set at 0.2 mm using the casting knife. After 15 s of exposure to the air, the glass plate with the casted solution was quickly immersed in a water bath and left for 24 hours to ensure complete phase inversion.

**Table 1:** Composition of Ag-GO polyamide 6,6 nanocomposite membrane.

Sample	Ag-GO (wt%)	Polyamide 6,6: Formic acid: Ag-GO
NY1	0.0%	1:4:0.000
NY2	0.2%	1:4:0.002
NY3	0.5%	1:4:0.005
NY4	0.8%	1:4:0.008
NY5	1.0%	1:4:0.010

### Membrane Characterization

The hydrophilicity of the membrane surface was represented by contact angle that was measured using Kruss GmbH FNY12MKE Easy Drop, Germany under ambient conditions. Fourier-transform infrared spectroscopy (FTIR) analysis was conducted using a Nicolet 6700 Thermo Scientific-FITR spectrometer (United States) to identify the functional groups present on the membrane. Membrane surface zeta potential was measured using the Malvern Surface Zeta Potential Cell, Malvern Instruments, UK. The zeta potential of membrane surface was measured in 0.1 mM NaCl at pH 7 using 300–350 nm latex particles as the tracer particles (DTS1235 Malvern UK).

The overall membrane porosity (ε) was calculated using the gravimetric method^29^, as defined in Equation (1):

1 $$\varepsilon =\frac{{\omega }_{1}-{\omega }_{2}}{A\times l\times {d}_{w}}$$

where ω_1_ is the wet membrane weight (g), ω_2_ is the dried membrane weight (g), *A* is the membrane surface area (m^2^), *l* is the membrane thickness (m), and d_w_ is the water density (998 kg/m^3^).

Using the porosity data and Guerout–Elford–Ferry equation^35,36^, the relative pore sizes of the fabricated membranes were calculated using Equation (2):

2 $${r}_{m}=\sqrt{\frac{(2.9-1.75\varepsilon )8\eta lQ}{\varepsilon \times A\times {\rm{\Delta }}P}}$$

where *η* is the water viscosity (8.9 × 10^−4^ Pa.s), *Q* is the volume of pure water permeated through the membrane per unit time (m^3^/s), and *ΔP* is the operational pressure (0.4 MPa).

### Membrane Performance Evaluation

#### Membrane Flux

Sterlitech^TM^ HP4750 dead end stirred cell was used to evaluate the flux of the fabricated polyamide membrane. In order to remove the residual chemicals within the produced membrane and to obtain a steadier solution flux, compaction was carried out for 20 min at a pressure of 5 bars. After the compaction step, membrane pure water flux was conducted for 120 min at a pressure of 4 bars.

The permeate flux was calculated using Equation (3):

3 $$J=(\frac{{\rm{\Delta }}v}{A{\rm{\Delta }}T})$$

where J is the measured permeate flux (L/m^2^ h), ΔV is the permeate cumulative volume (L), A is the effective membrane surface area (m^2^), and ΔT is the filtration duration (h).

#### Membrane Fouling Analysis and Rejection Calculation

**Figure 1:**
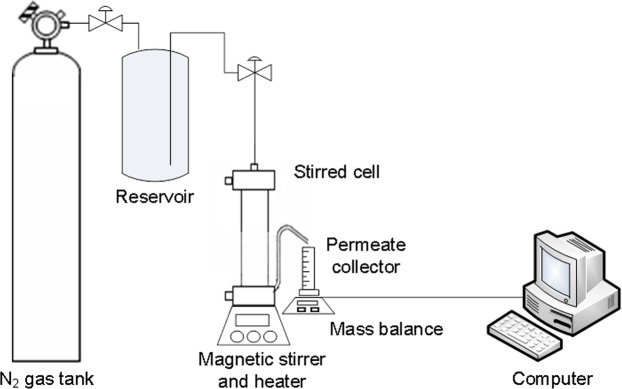
Schematic diagram of stirred cell system used in this study.

After the water flux tests, a permeation experiment was also carried out using the Sterlitech stirred cell with 3 g/L BSA solution at 4 bars. A reservoir filled with 1 L of BSA solution at 3 g/L was connected to the stirred cell to continuously refill the stirred cell throughout the filtration process (Fig. 1). A constant feed solution concentration of 3 g/L has been assumed in this study by averaging the bulk solution concentration. A constant filtration duration of 150 min was set in this study, and a mean concentration of permeate solution was then measured. Variation in solute concentration from the bulk solution to membrane surface during the filtration process was not directly measured in this study because it will involve complicated analyses and sophisticated instruments that can perform *in situ* measurement. Accumulation of foulants on the membrane surface, however, can be dynamically represented by continuous solution flux measurement. The flux variation profiles in this study have been represented by normalized flux by taking the pure water flux at the same operating condition as the basis of comparison. BSA was selected for this fouling test because it was one of the most common foulants used in membrane performance testing^37,38^. The normalized flux (F_n_) was measured for 150-minute filtration period based on the ratio of flux for BSA solution (j_BSA_) to pure water flux (j_w_). Flux recovery ratio (FRR) was calculated based on the ratio of recovered ultrapure water flux after BSA solution (j_w2_) to pure water flux (j_w_). Afterwards, the membranes were rinsed for 15 min using ultrapure water. Next, the membrane permeation flux with ultrapure water was conducted again. The total fouling ratio (R_t_), reversible fouling ratio (R_r_) and irreversible fouling ratio (R_ir_) of the membranes were measured using Equations (4–8) to analyse the overall fouling pattern:

4 $$FRR=(\frac{{j}_{w2}}{{j}_{w}})\times 100$$

5 $${R}_{t}( \% )=(1-\frac{{j}_{BSA}}{{j}_{w}})\times 100$$

6 $${R}_{r}( \% )=(\frac{{j}_{w-}{j}_{w2}}{{j}_{w}})\times 100$$

7 $${R}_{ir}( \% )={R}_{t}-{R}_{r}$$

8 $${F}_{n}=(\frac{{j}_{BSA}}{{j}_{w}})$$

where j_BSA_ is the flux for BSA solution, j_w_ is pure water flux, and j_w2_ is the recovered ultrapure water flux after BSA filtration.

In addition to BSA (3 g/L), Congo red (0.1 g/L) was also chosen as the foulant for rejection test due to its presence in various industrial effluents such as textiles, printing, dyeing, paper, and plastic^39^.

Membrane rejection capability for Congo red and BSA was calculated using Equation (9):

9 $$R=1-\frac{{C}_{p}}{{C}_{f}}\times 100 \% $$

where C_p_ and C_f_ are the concentrations of permeate and feed solutions, respectively.

#### Membrane Stability Test

After each filtration test for 5 hours, the filtrate water was collected and sent to ICP-MS (Elan 9000 PerkinElmer, USA) analysis to detect the presence of Ag. The presence of Ag in the filtrate will reflect the stability of Ag in the membrane matrix.

#### Membrane Tensile Strength

The tensile strength of the membranes was analysed using a CT texture analyzer from Brookfield Engineering USA. 25 mm × 75 mm membrane samples were inserted into the grips fitted with rubber to maximize the contact adhesion with samples. Tension test trigger, deformation and speed were set at 0.1–100 g, 0.1–101.6 mm and speed to 0.01–10 mm/s, respectively. Membranes were dried and tested at ambient conditions.

#### Zeta Potential Measurement

Surface Zeta potential was measured using the Malvern Surface Zeta Potential Cell. The zeta potential of PES membrane surface was measured in 0.1 mM NaCl at pH 7 using 300–350 nm latex particles as the tracer particles (DTS1235 Malvern UK).

#### Membrane Antibacterial Analysis

Membrane antimicrobial analysis was conducted using *Escherichia coli* (*E. coli*) as a model bio-foulant^40^. *E. coli* were first cultured in a nutrient broth. After the growth of stock solution achieved 109 × 10^7^ CFU/mL (OD600 1–1.5), the *E. coli* were serially weakened to 20 × 10^7^ CFU/mL from the stock using nutrient broth medium^41^. After this stage, the fabricated membranes were soaked in the diluted *E. coli* solution for 10 min. They were then placed on the nutrient agar plates and incubated at 35 °C overnight. The colony forming units (CFUs) on the membrane surface were then observed using FESEM.

## Results and Discussion

### Characterization of Ag-GO nanoplates

**Figure 2:**
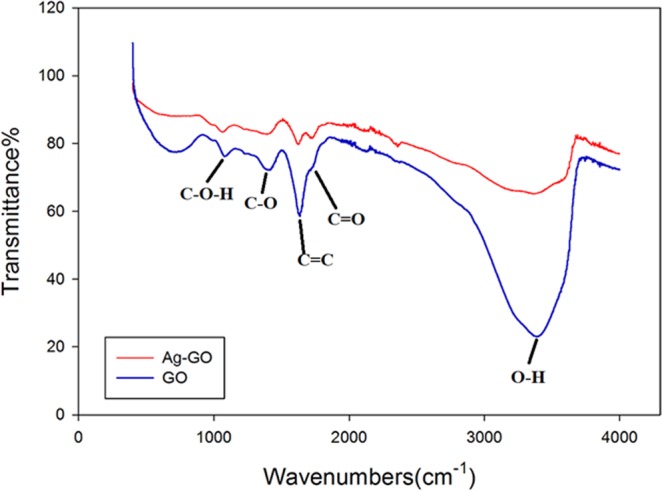
FTIR spectroscopy of GO and Ag-GO.

**Figure 3:**
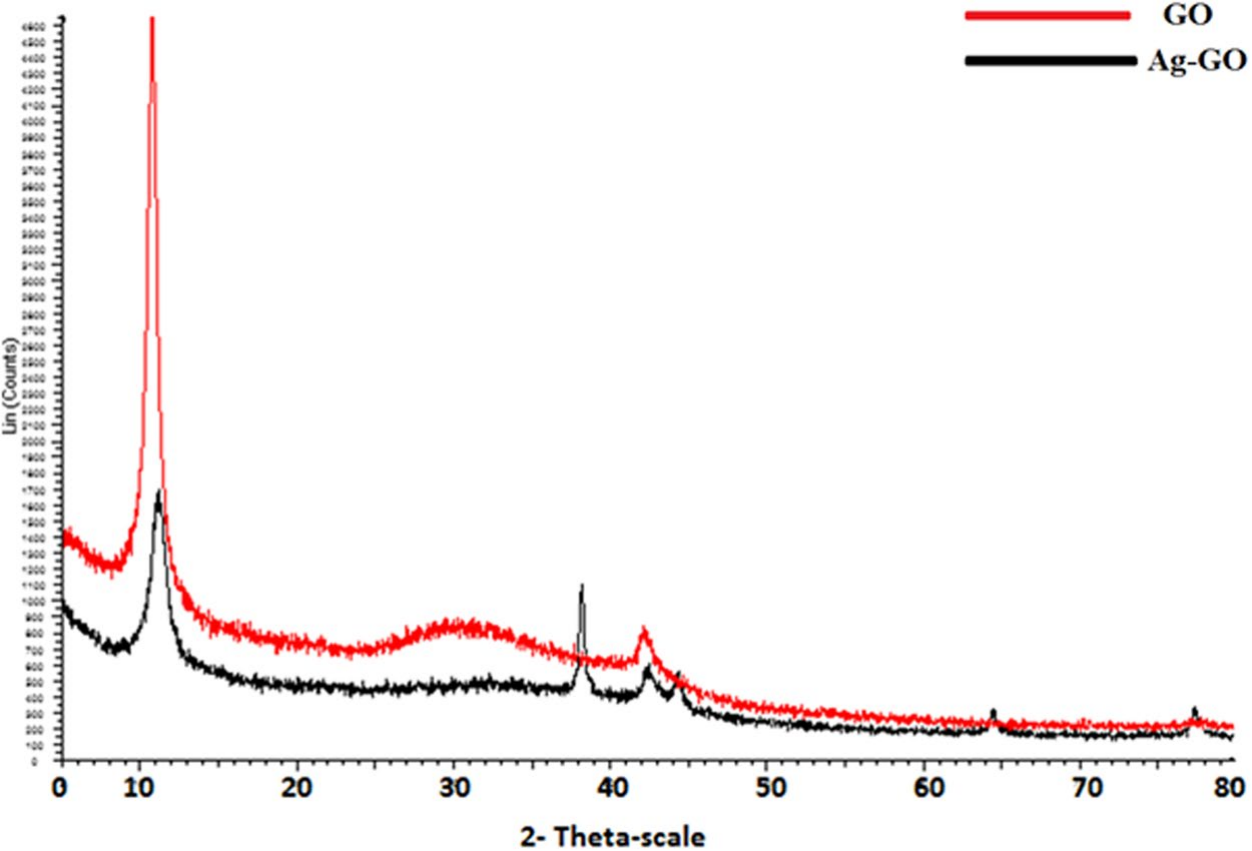
XRD patterns of GO and Ag-GO nanoplates.

**Figure 4:**
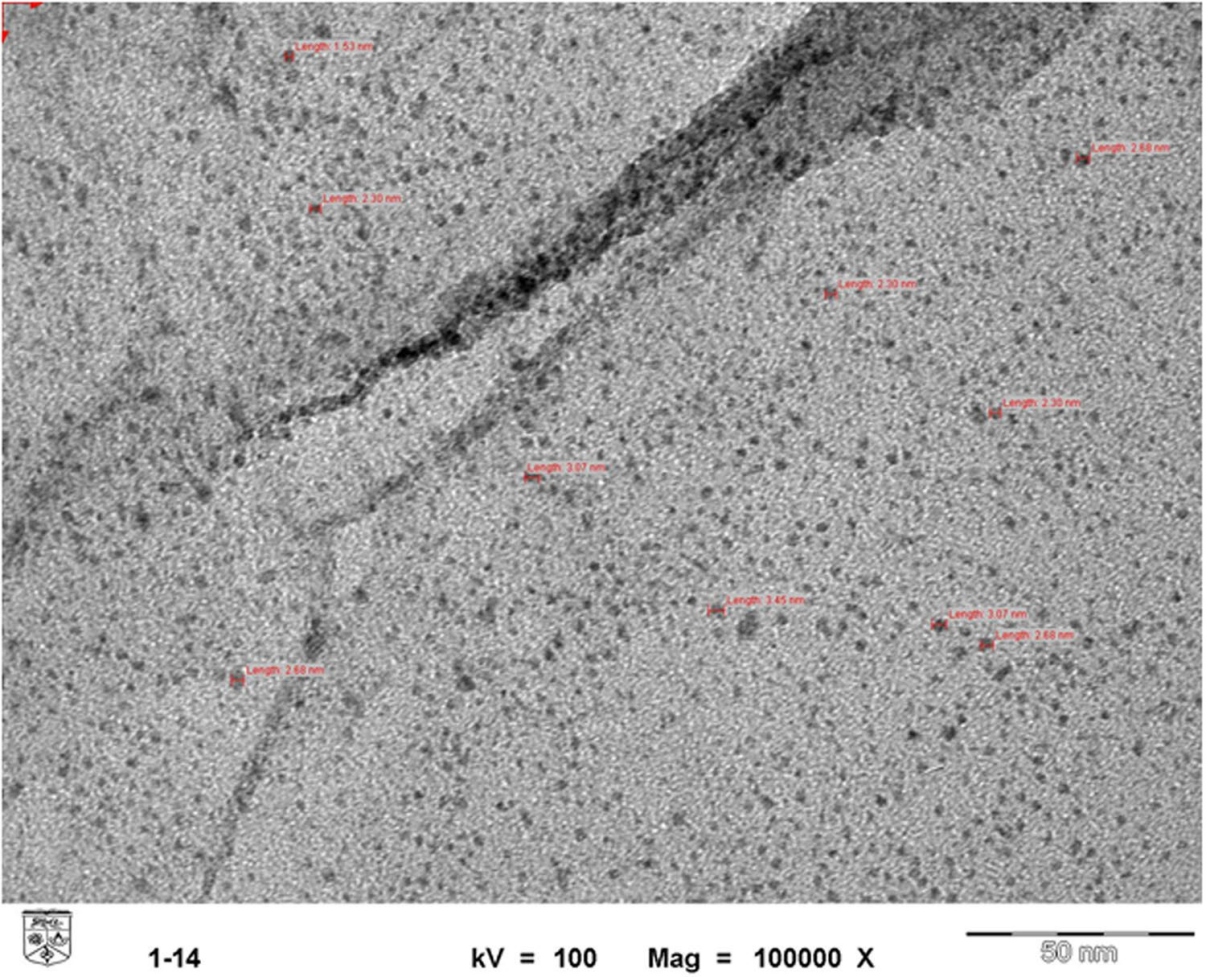
TEM micrograph of Ag-GO nanoplates.

Figure 2 demonstrates the FTIR spectroscopy of the synthesized GO and Ag-GO. The sharp peak at 1725 cm^−1^ and broad peaks around 3363.1 cm^−1^ and 1302 cm^−1^ can be seen in both GO and Ag-GO, which correspond to the stretching vibration and deformation vibration of O–H groups^42^. The two bands at 1066 cm^−1^ and 1200 cm^−1^, originally from the C=O stretching vibrations of alkoxy groups were seen in the spectrum too. These data supported the existence of carboxylic acid groups on the GO surface. The FTIR spectrum of Ag-GO pointed out all the oxygenated functional groups of graphene oxide. However, an absolute decrease in the absorption intensity of the functional group bands has been observed for the Ag-GO sample. This decrease could be due to the shadowing effect of the silver nanoparticles decorated on the GO nanoplates^43^, as supported by XRD analysis. The XRD pattern for the Ag-GO (Fig. 3) showed peaks at about 38, 44, 64 and 77°, whereby these peaks can be assigned to the Miller indices of (111), (200), (220) and (311) crystallographic planes of face-centred cubic (fcc) of Ag nanoparticles, respectively^44^. Indeed, TEM micrograph in Fig. 4 shows that the silver nanoparticles (with an average size of 4 nm) were uniformly distributed across the GO sheet. Such observation indicates that agglomeration phenomenon did not occur in this case. Hence, the decoration of Ag nanoparticles on GO nanoplates has successfully resolved the issue of Ag agg

### Membrane characterization

**Figure 5:**
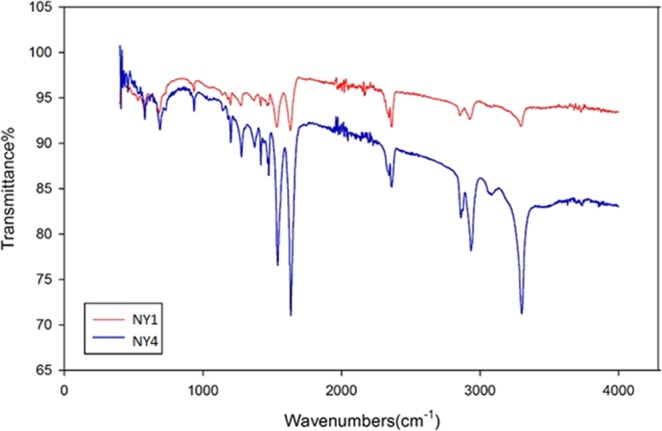
FTIR spectra of pure polyamide 6,6 (NY1) and Ag-GO/polyamide 6,6 (NY4).

Figure 5 displays the functional groups present in polyamide 6,6 and Ag-GO composite membranes. According to the FTIR spectra, the vibrational frequencies of peaks for both polyamide 6,6 and Ag-GO/polyamide 6,6 samples are determined and listed in Table S1 (Supplementary Document). The spectra obtained through FTIR analysis confirmed the standard footprints of polyamide 6,6^1^. The frequency bands of polyamide 6,6 and Ag-GO/polyamide 6,6 show some slight shifting (between 3–9 cm^−1^) after mixing with Ag-GO. The band shift exhibited that the Ag-GO has been successfully embedded into polyamide 6,6 matrixes, in which all peak intensities were reduced^2^.

**Figure 6:**
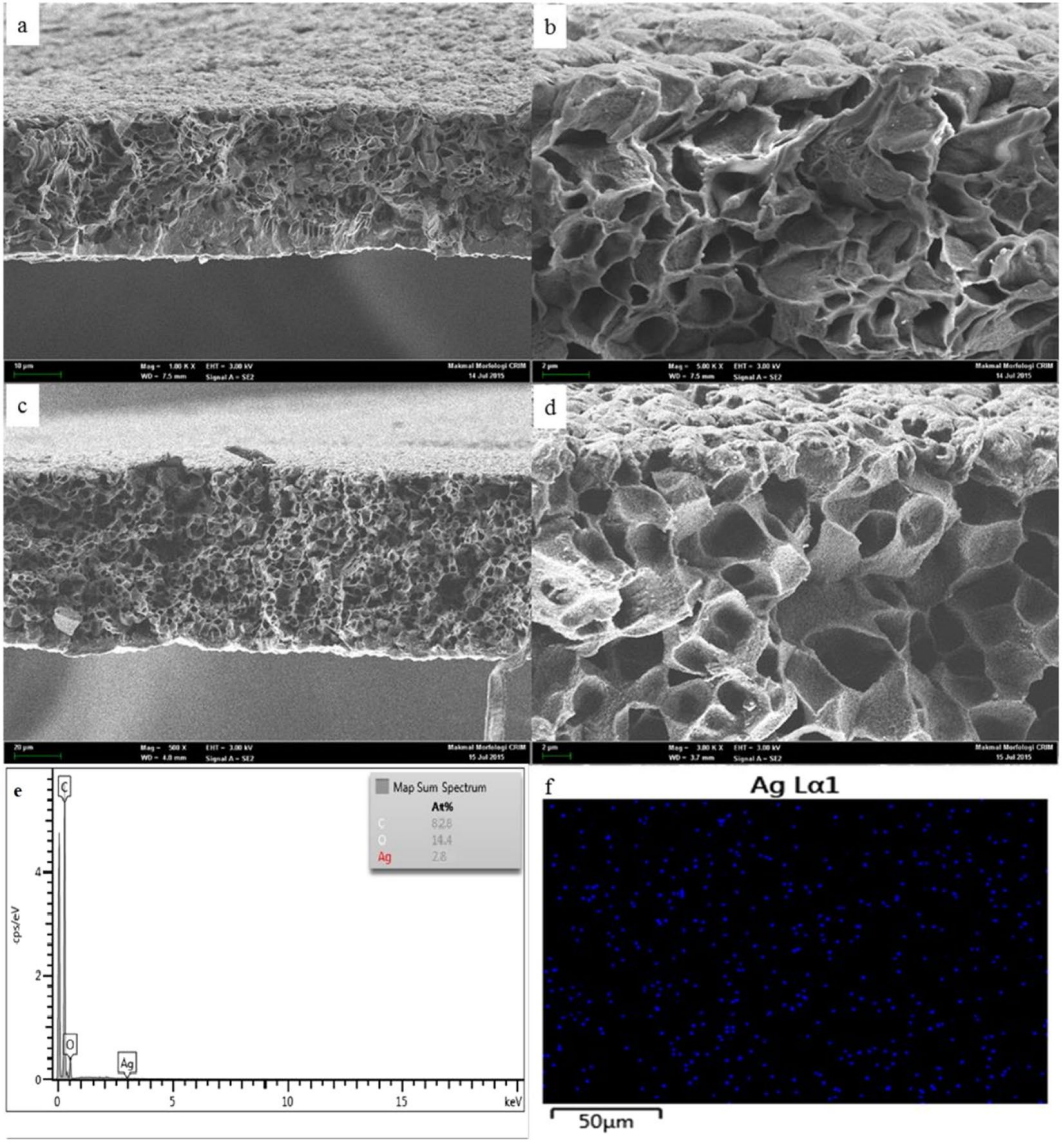
Cross-sectional FESEM images of membranes: (**a**) 500X (**b**) 3kX for Pure polyamide 6,6 membrane (NY1); (**c**) 500X (**d**) 3kX for Ag-GO/polyamide 6,6 membrane (NY4); (**e**) EDX spectrum of NY4 membrane; (**f**) Mapping of NY4 membrane.

**Figure 7:**
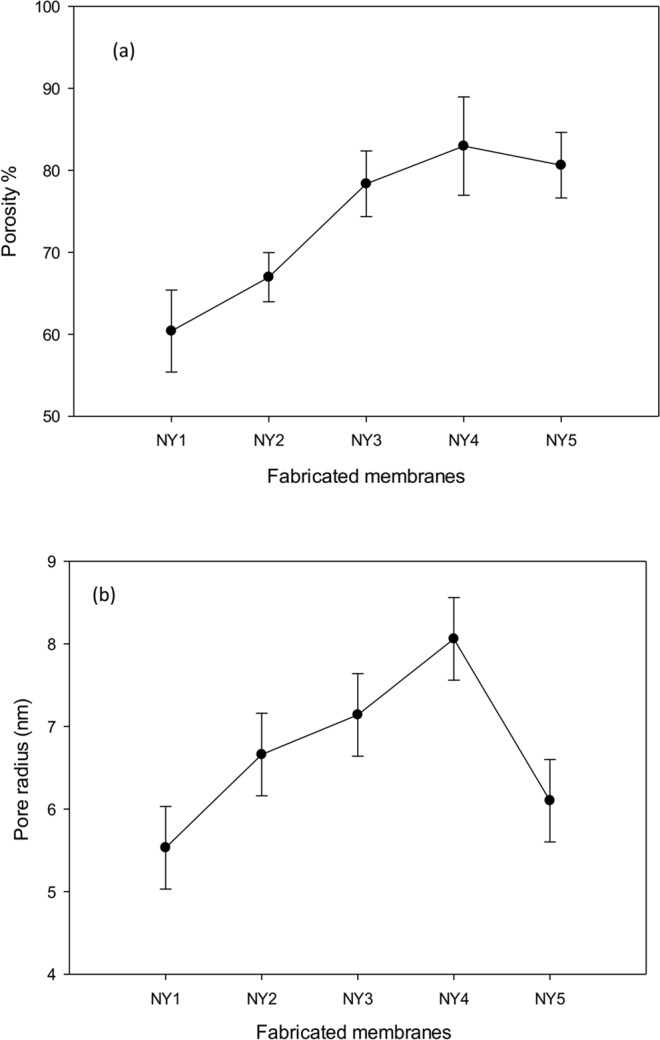
(**a**) Porosity and (**b**) pore size of fabricated membranes.

The cross-sectional structure of the membranes (Fig. 6) presents a sponge-like structure for all the fabricated membranes. It can be observed that the membranes embedded with Ag-GO are more porous compared to the pristine polyamide 6,6 membrane. The addition of Ag-GO induced a high porosity structure. This development is favourable to the membrane permeability as it was generally reported that membrane with higher porosity recorded higher water flux^29^. In order to determine the distribution of silver nanoparticles in the matrix of the membranes, the EDX analysis and mapping mode has been employed. Figure 6(e) illustrates the EDX image of the membrane with 0.8 wt% of Ag-GO (NY4). The existence of the silver nanoparticles can be proven by the peaks located at 2.65 and 2.984, while the EDX mapping results presented in Fig. 7(f) showed a perfect distribution of the silver nanoparticles in the matrix of polyamide membrane. This clearly shows that Ag-GO nanocomposite was nicely embedded into the polyamide 6,6 polymer matrix without agglomeration issue.

The overall porosities and the pore radius of the membranes can be summarized through the observation in Fig. 7(a). The fabricated membranes exhibited porosities in the range of 60 to 80%, which are greatly desirable for the polyamide 6,6 membranes. The modified membranes showed higher porosities compared to the pure polyamide 6,6 membranes, supporting the physical observation from FESEM in Fig. 6, which could be ascribed to the addition of Ag-GO nanoplates to the membrane matrices^29,36,45^. The pore radius of the fabricated membrane as presented in Fig. 7(b) revealed that the initial addition of Ag-GO increases the pore radius while the addition of 1 wt% of Ag-GO reduces the size of pore radius. This phenomenon can be related to the viscosity of casting solution and mass transfer rate between the phases as reported by previous studies^46^. Coincidently, similar pore radii also can be obtained using Hagen-Poiseuille equation by assuming the cylindrical length as membrane thickness (0.2 mm). Ag-GO nanoplates have been reported to be one of the most hydrophilic derivatives of graphene-family^30,31,43,47,48^. The addition of hydrophilic nanoplates to the casting solution can decrease the thermodynamic stability of the casting solution (weakening the van der Waals interaction between the polymer and solvent molecules). This enabled the solvent molecules to diffuse rapidly from the polymer matrix to the coagulation bath and thus resulted in the formation of larger pore size and porosity structure of the polymer membrane^28^.

**Figure 8:**
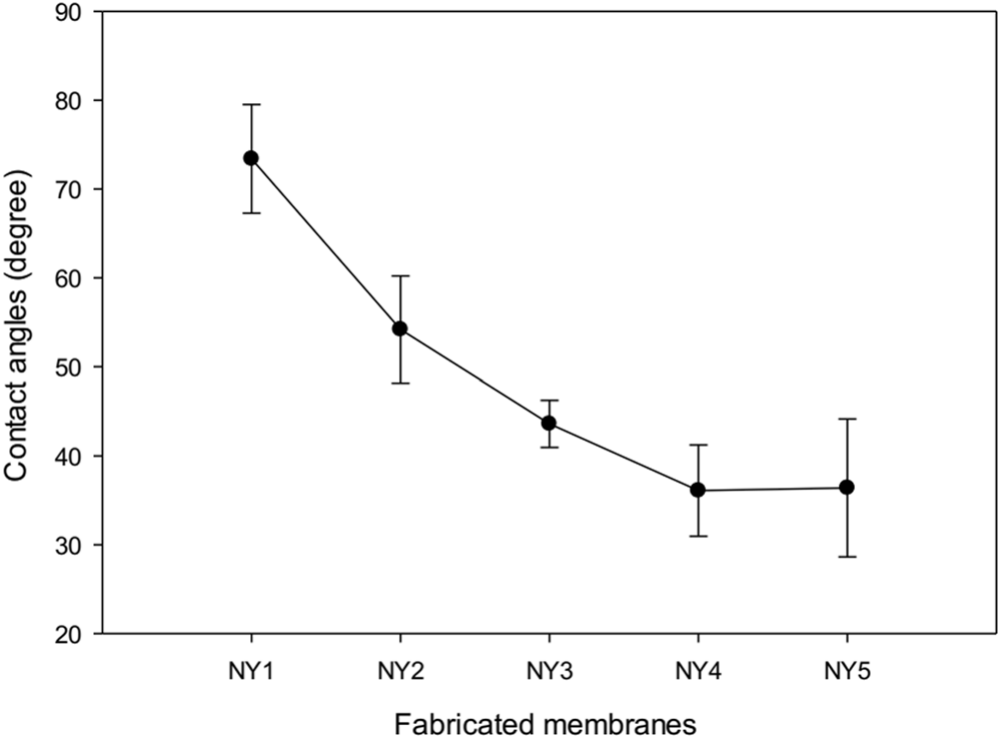
Contact angles of fabricated membranes.

As illustrated in Fig. 8, the addition of Ag-GO to the polyamide membrane has a direct impact on the contact angle reduction of the fabricated membranes. The pristine polyamide 6,6 membrane recorded the highest contact angle of 73.4 ± 6.12°. The addition of 0.2 to 1 wt% of Ag-GO reduced the water contact angle to 54.2 ± 6.03°, 43.6 ± 0.65°, 36.1 ± 5.14° and 39.4 ± 7.76°, respectively. The hydrophilicity of the membrane can be indicated by its surface wettability. The presence of epoxy, carboxyl, and the hydroxyl functional groups of the GO and the synergic effect presence of Ag-GO in the membrane structure can lead to the increment of interface energy in the polyamide membranes^28^. Consequently, the adhesive forces between the water molecules and the membrane surface will be higher than the cohesive forces exist between the molecules of the water. Thus the water molecules will be attracted to the surface of the membrane rather than to each other, resulting in lower contact angle (better hydrophilicity)^49^.

**Figure 9:**
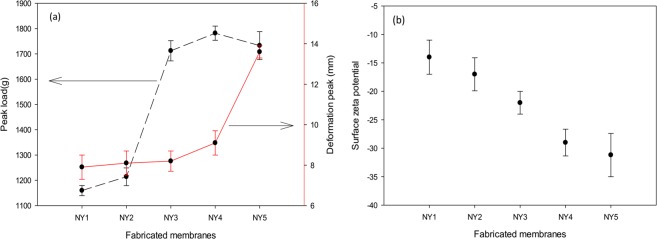
(**a**) Tensile strength of fabricated membranes. (**b**) Surface zeta potential (mV) of fabricated membranes.

Apart from the improvement of hydrophilicity, Fig. 9(a) shows the tensile strength test of the fabricated membranes where a significant mechanical strength enhancement was observed. The peak load for the pure polyamide membrane was 1159.5 g, and the deformation peak was at 7.9 mm. On the other hand, the peak load for the NY 5 membrane (1% of Ag-GO) was at 1733 g, and the deformation peak was at 13.6 mm, with an increment of 39.69% in mechanical strength. This phenomenon could be attributed to the attractive forces between the Ag-GO nanoplates structure and the polyamide 6,6 membrane, resulting in the formation of sea-island structure between polyamide 6,6 and Ag-GO^50^. Homogeneous dispersion of Ag-GO in the matrix of membrane resulted in efficient and uniform load transfer throughout the membrane matrix and thus increasing the mechanical properties of the modified membranes. In a nutshell, the enhanced mechanical properties were due to the uniform dispersion of Ag-GO nanoplates and effective stress transfer^50,51^.

Figure 9(b) illustrates the surface zeta potentials of the fabricated membranes. The addition of the Ag-GO to the structure of the membrane propels the charge towards more negative range, changing from −14 ± 6 to −31 ± 3.8 (mV) for pure membrane and membrane with 1 wt% of Ag-GO, respectively. This phenomenon could be attributed to the addition of negatively charged Ag-GO (−47.4 mV at pH 7) to the membrane matrix^52^, and could be favourable for biofouling prevention as the modification of the polymer surface charge has been proven to be an effective mean of biofilm prevention^53,54^.

### Membrane Performance Evaluation

**Figure 10:**
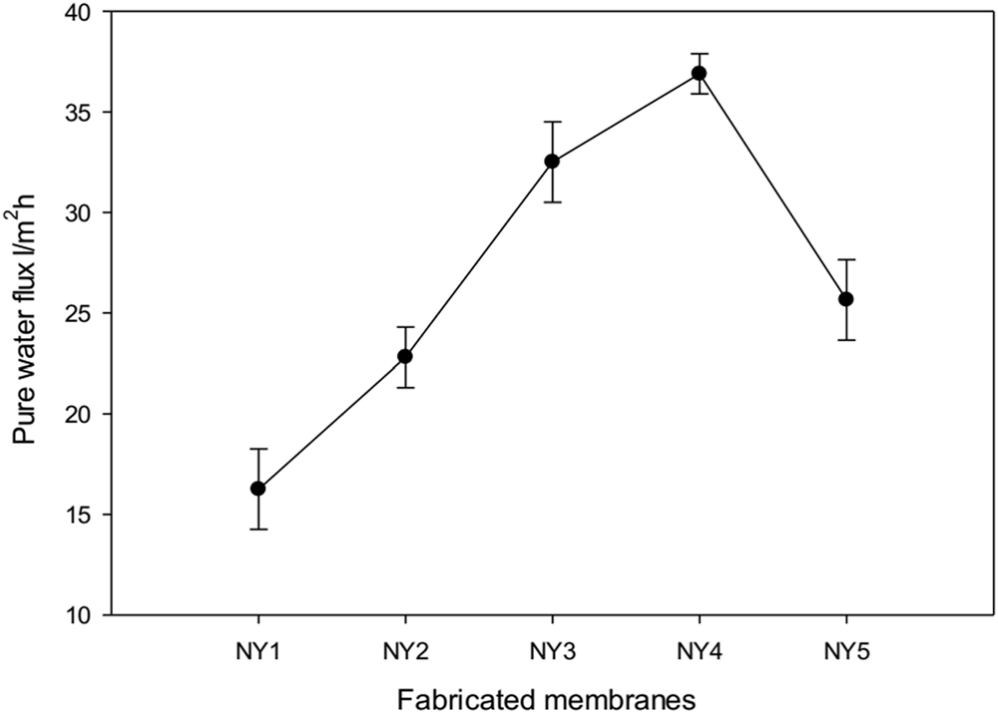
Pure-water flux of fabricated membranes.

Overall, the incorporation of Ag-GO nanoplates has improved the membrane properties, and the issue of nanoparticles agglomeration was resolved by the decoration of Ag onto GO nanoplates. Figure 10 shows that the membrane pure water flux is linked directly with hydrophilicity and porosity structure of the fabricated membranes (Figs 7 and 8). The incorporation of 0.8 wt% Ag-GO in the matrix of the membrane (NY4) resulted in the highest water flux, corresponding with the lowest contact angle and largest pore radius. Addition of nanoplates in the membrane matrices in this study increased the membrane porosity, which could be explained with the presence of holes in nanoplates and formation of nanochannels on membrane surfaces as observed in the SEM image on Fig. 6. This could be one of the reasons for higher membrane fluxes as observed in this study^55^. On the other hand, the membranes incorporated with 1 wt% (NY5) of the nanoplates recorded a lower flux. The addition of 1 wt% might increase the viscosity of the casting solution and thus slow down the mass transfer between the solvent and non-solvent phase. The slow mass transfer between the solvent and non-solvent phase causes the formation of smaller pores size (Fig. 7b) as explained in the previous section. This phenomenon was in accordance with studies reported by previous authors^18,19,23,56,57^.

### Membrane Stability Tests

The results from ICP-MS of the filtrate water show no trace of silver in the permeate (Table S2 in the Supplementary Document), proving that the silver did not leach out of the membranes after 5 hours of filtration. As reported by other authors, pure silver nanoparticles have the tendency to leach out from the membrane. However, this phenomenon did not occur in the current study, showing that GO provided numerous anchor points for the attachment of Ag nanoparticles^24,58^.

### Fouling and Rejection analysis

**Figure 11:**
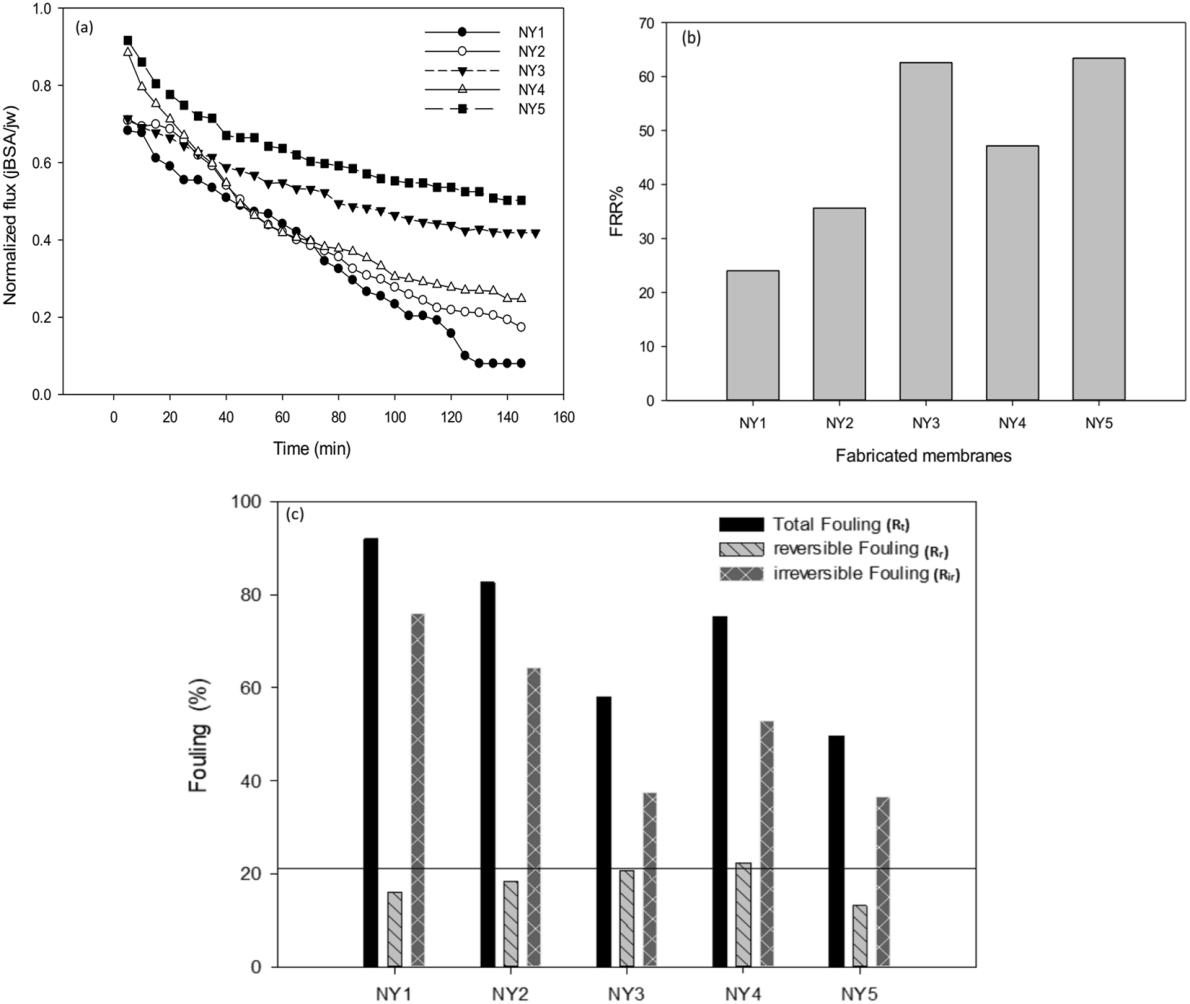
(**a**) Flux decline profiles of fabricated membranes, (**b**) Flux recovery ratio of fabricated membranes (FRR%), (**c**) Fouling profiles of fabricated membranes.

The flux performance for BSA filtration process is presented in Fig. 11(a). From this figure, it can be concluded that the fouling tendency (reflected by the steepness of flux decline) of the membrane decreases with the increase of Ag-GO ratio in membrane matrix. However, NY4 is an exception. This might be due to the fact that the foulant particles can easily penetrate and clog the larger membrane pores appear on NY4 membrane^59^. The flux recovery ratios (FRR) of membranes are depicted in Fig. 11(b). In general, a high FRR% indicates a better fouling resistance for the membrane. It is obvious that the FRR% of the modified membranes is higher than the pure membrane (~25% to ~60% respectively). This indicates that the blending of Ag-GO increased the organic fouling resistance of the membranes. The highest FRR% was related to NY5 membrane (63.38%) while lowest to NY1 membrane with 24.1%. It is well established that hydrophilic surface can adsorb water molecules and form a water layer on the membrane surface, which retards the absorption of protein and other fouling agents to foul the membrane^19^. The trend that was observed for the flux recovery ratio is matched by the contact angle reduction of the membranes. However, the NY4 membrane is an exception to this trend due to the larger pore radius, possibly caused by the protein that might be able to penetrate into the pores and cause the fouling. It could be seen that the effect of the pore size overwhelmed the improvement in terms of surface charge (Fig. 9) and hydrophilicity.

Furthermore, Fig. 11(c) shows the overall fouling tendency of the membranes. The total fouling of the mixed matrix membranes was considerably reduced from 92% for NY1 to 49.72% for NY5. Reversible and irreversible fouling of the membranes is also demonstrated in Fig. 11(c). These results showed that the modified membranes displayed remarkable antifouling properties in comparison to the pristine polyamide 6,6 membrane. The irreversible fouling of the polyamide 6,6 membrane was reduced from 76% to 37% (NY5). The improvement could be attributed to the more negatively charged and hydrophilicity of the NY5 membrane that helped to repel the foulant from persistently adsorbed to the membrane surface^1^. This exhibits that reversible fouling was the dominant phenomenon in total fouling for the modified membranes and hydraulic cleaning would easily reverse the fouling effect. The phenomenon is attributed solely to the fact that a low percentage of the nanoplates were uniformly and perfectly distributed across the membrane matrix providing enhanced surface properties such as charge and hydrophilicity that resulted in lower overall fouling. Thus, this proves that the addition of small amount of Ag-GO to the membrane helped to alter the membrane surface properties and enhanced the overall properties of the fabricated membrane.

**Figure 12:**
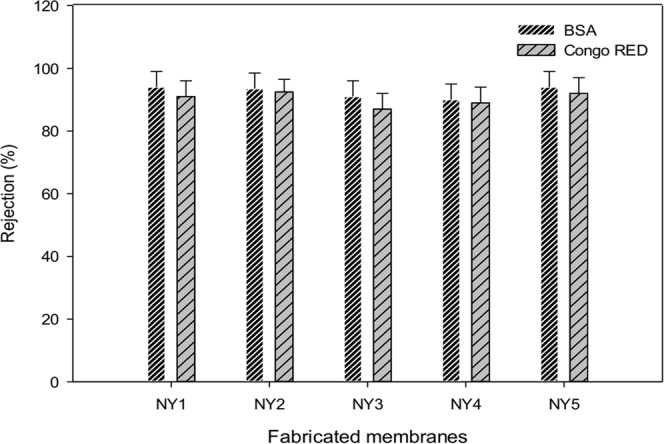
Rejection of Congo red and BSA for fabricated membranes.

According to Fig. 12, the rejection percentage of Congo red is relatively high (89% to 92%) despite the fact that the pore size and water flux of the modified membranes increased with the addition of Ag-GO. This phenomenon was possibly attributed to the hydrophilicity enhancement of the modified membranes. The establishment of highly hydrophilic membrane structure enhances the affinity of membrane surface to water molecules rather than to Congo red, resulting in lower hydraulic resistance. On top of that, the negatively charged surface of the membrane might repel the negatively charged Congo red too^60^. Hence, the change in pore size and water flux does not affect the rejection dramatically.

On the other hand, similar findings were observed for BSA rejection tests. All the modified membranes rejected more than 90% of BSA. As shown in Fig. 12, the addition of 0.8 wt% and 1 wt% of Ag-GO would result in even better BSA rejection, approaching 95% and 97%, respectively. The rejection capabilities of the membranes were not compromised, even though the modified membranes have higher flux and bigger pore radius. This could be attributed to the fact that the modified membranes have higher hydrophilic surface and negative charge that could minimize the attachment of BSA on the membrane surface^35,61,62^.

Looking at both sets of rejection data, it can be concluded that the rejection capability of polyamide 6,6 membranes is thought to occur in few different mechanisms. Firstly, sieving (steric) rejection that depends on the pore size. However, the addition of the graphene oxide increased the pore size of the membrane and porosity simultaneously. The increase in pore size and porosity contributed to higher membrane fluxes. Surprisingly, the rejection of both pollutants were not compromised much from larger pore size and higher flux. This indicated the role played by other factors that overwhelmed the negative effect of larger pore size on the rejection. The interactions between Ag-GO and polyamide made the membrane more hydrophilic (as can be seen by the decrease in contact angle), which would reduce the adsorption of organic pollutants on the surface of the membrane. Thus, it reduced the tendency of foulants to permeate through the membrane. The establishment of highly hydrophilic membrane structure could increase the affinity of the membrane to water rather than the pollutant, resulting in lower hydraulic resistance. Besides, the enhanced rejection properties could be correlated with the zeta potential of the membrane. The negatively charged membranes would cause Donnan repulsion to the pollutants that prevent it from permeating through the membrane. The synergistic effect of surface hydrophilicity and charge repulsion resulted in the observed membrane performance, where the rejection capability was not compromised even though at higher flux.

### Antibacterial Performance

**Figure 13:**
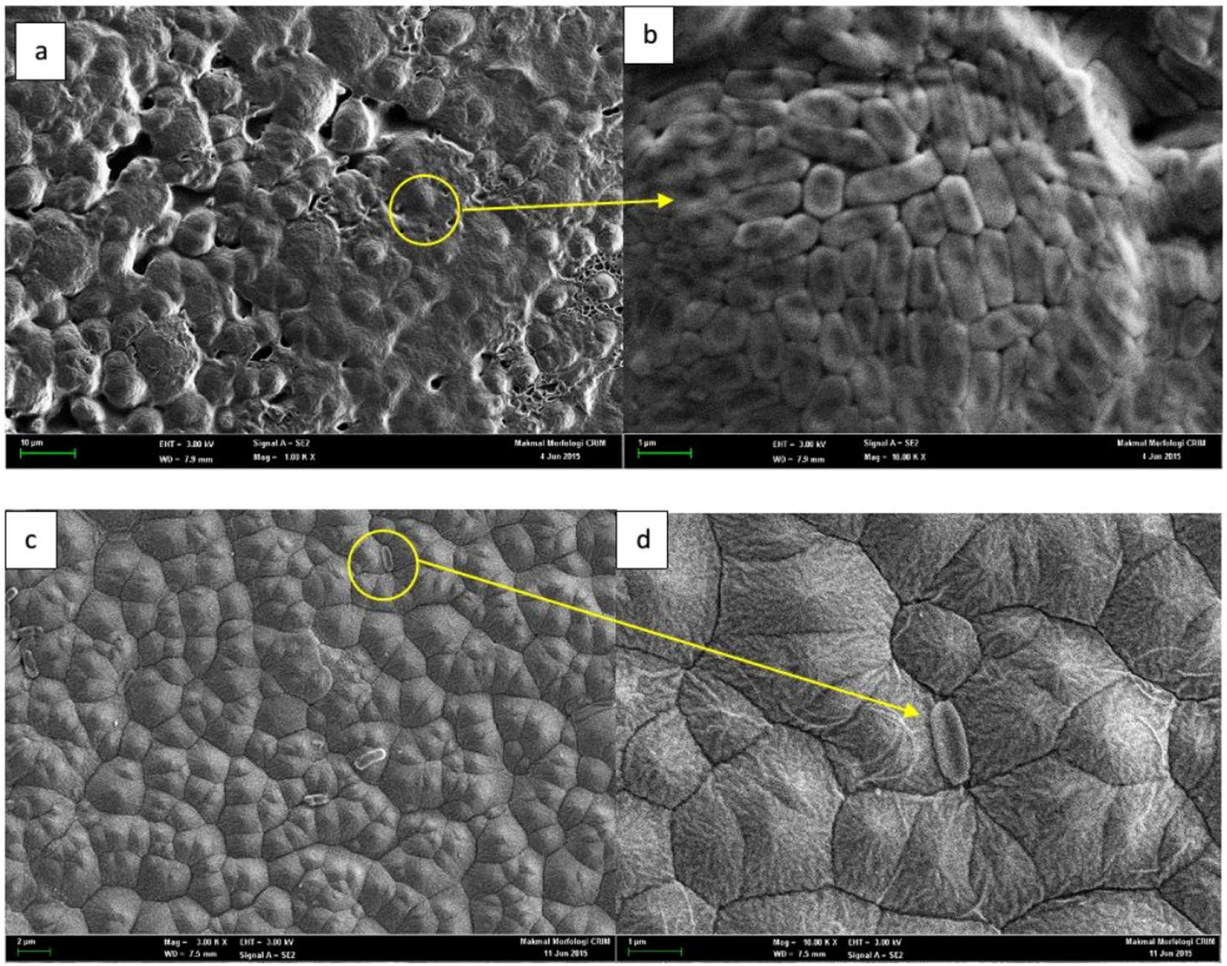
FESEM images of the membrane under antimicrobial test: Pure polyamide 6,6 membrane (**a**). NY1, 3kx; (**b**). 10kx; Ag-GO/polyamide 6,6 membrane (**c**). NY4 3kx; (**d**). 10kx.

Figures 13(a,b) illustrates the FESEM images of the pristine polyamide 6,6 membrane after the antibacterial test. Theoretically, surfaces with higher hydrophobicity are more immune to bacterial growth, as the microbial adhesion to the surface will be more difficult^63,64^. However, it can be seen that the surface of the pristine polyamide 6,6 membrane is covered by bacterial colonies. *E. coli* grew into colonies and formed a layer that completely covered the pores of the pure polyamide 6,6 membrane^65^.

In contrast, the addition of Ag-GO nanoplates to the membrane stopped the colonial growth of the bacteria. Figures 13(c) and 12(d) show the FESEM image of the membrane containing 0.8 wt% of the Ag-GO nanoplates. There was no colony formation or bacterial growth of the surface of the membrane, proving that the decoration of silver nanoparticles of graphene oxide is an effective way of biofilm formation control. As aforementioned, the membrane surface showed a more negative charge when embedded with Ag-GO. The synergetic effect of static antimicrobial of GO and oligodynamic antimicrobial of silver attributed to the production of a membrane that successfully restricted the growth of gram-negative *E. coli* despite the fact that *E. coli* shows resistive towards most antibiotics^66^.

### Benchmarking with Past Studies

The findings from this study were benchmarked against other similar studies on the issues of nanoparticles agglomeration, leaching and amount of nanoparticles used in the synthesis of membrane. It is not possible to draw a solid correlation between the amount of Ag used in the study and the performance improved by the incorporation of nanoparticles. However, the best performing membrane in this study only employed 0.2 wt% of pure silver, which is much lower than most of the amount reported in other literature^24,67–75^. In this study, flux improvement of 133% and irreversible fouling reduction of 40% were observed while the rejection maintained at the same level.

There are several literatures reported on the leaching of Ag after filtration as reported in Table 2. It was discovered that all the studies with leaching issues did not decorate the Ag nanoparticles on any medium. The lack of strong anchor sites for Ag nanoparticles could be the reason that contributed to the leaching issue. Such a phenomenon was not observed in this study as the GO provided a better anchor point for the Ag nanoparticles.

**Table 2:** Membrane synthesis with various silver nanoparticles loadings.

NP %	Polymer	Significant Findings	Leaching	Flux and rejection% enhancement	Ref.
1 × 10^−3^, 1 × 10^−2^, 1 × 10^−1^ mol/dm^3^	PES hollow fibre; (30 kDa; 150 kDa) grafted with acrylamide	Exhibited improved organic antifouling properties with BSA solution and antibacterial properties with *E. coli*	NA	-25% enhancement in pure water flux, -40% better BSA rejection	^67^
0.25, 0.5, 1.0% w/w	PSf (22 kDa), 18% w/w; PVP 2% w/w	Improved the protein (BSA) and carbohydrate (dextran) filtration performances as well as biofouling performance with real activated sludge filtration	Investigated using ICP, membrane showed silver leaching	-12% enhancement for 0.25% -58% decreased in pure water flux for 1% due to agglomeration	^68^
Biogenic nano-silver, (Bio-Ag^0^-6), 0.1, 0.3, 0.5, 1.0 wt%	PES UF membrane	Improved the water permeability and fouling for BSA solution; Excellent antibacterial activity of membrane towards *Escherichia coli* & *Pseudomonas aeruginosa*	Investigated using ICPMS, membrane showed silver leaching	-75% enhancement in pure water flux for 1% of Bio-Ag^0^, -agglomeration was reduced	^69^
Biogenic AgNPs, (Bio-Ag^0^-6); Chemical AgNPs, (10 g/L)	PSf 17.5 wt%; Thin film composite NF membrane; PVP 0.5 wt%	Enhanced the hydrophilicity and water flux, while maintaining good salt rejection (Na_2_SO_4_); Effective biocide agent to mitigate TFC membrane biofouling	Investigated using ICPMS, membrane showed silver leaching	-40% enhancement in pure water flux	^70^
Ag^+^ ion, (5, 20, 40 mM)	PSf UF membrane; immobilized with polydopamine (PDA)	High permeation flux; better antifouling performance with BSA filtration; high antibacterial property for *E. coli* & *B. subtilis*.	NA	-36% enhancement in pure water flux, −10% enhancement for BSA rejection	^71^
0.1 M AgNO_3_	Polyimide (Torlon 4000 T polyamide-imide); 8 wt%	Superior performance of antifouling effect with BSA; inhibitory and biocidal properties against *E. coli* or *S. aureus*	Investigated using atomic adsorption spectrometer, showed silver leaching	-decreased in pure water flux	^72^
0.05 w/v% in TMC/hexane	PSF 15 wt%; thin-film nano-templated (PDA) composite NF membrane	Enhanced the separation performance (doubled water permeability, increased salt rejection to NaCl & MgSO_4_, enhanced NaCl/MgSO_4_ selectivity) and antimicrobial properties on *B. subtilis & E. coli*	Investigated using inductive coupled plasma optical, emission spectrometer, membranes showed silver leaching,	-110% enhancement in pure water flux, -Agglomeration of silver nanoparticles was observed	^73^
0.5, 1.0, 1.5, 2.0 wt% Ag	PVC 17 wt%; hollow fibre UF membrane	Enhanced antifouling properties and COD removal (influent wastewater from the pharmaceutical company) with good antibacterial properties (*E. coli*); suitable application in industrial MBR	NA	-423% enhancement in pure water flux, -30% better COD removal	^74^
0.1 To 4% Ag, 70 nm, 0.1 to 4% Ag 30 nm	PSF 16% PVP 4%, flat sheet UF membrane	Effect Ag nanoparticle size on properties of the membrane was investigated. Pore size, hydrophilicity and flux of PS membrane was influenced by size of the Ag nano particles. In general, Ag with size of 30 nm showed better results. Membranes showed improved rejection of BSA	Investigated using atomic adsorption spectrometer, Silver with smaller size leached out faster	-150% enhancement in pure water flux for Ag with 30 nm, -100% enhancement in pure water flux for Ag with 70 nm, -30 and 25% better BSA rejection respectively	^24^
0.22 wt% Ag	15% PSf, 10% PVP, flat sheet UF membrane	antimicrobial activity against *E. coli* K12 and P. Mendocino KR1, and improvement in virus removal, Enhancement in terms of hydrophilicity and flux	Proved using ICPMS and TEM, membrane showed silver leaching	-30% enhancement in pure water flux	^27^
Ag-GO, 0.1, 0.3, 0.5, 0.8, 1 wt%	PSF, 18 wt% flat sheet UF membrane	This study adopted Ag-GO nanoparticles for the fabrication of the membrane for the first time. Anti-microbial test against *E. coli* showed that the membranes has a great resistance against bacterial growth.	NA	-55% enhancement in pure water flux Contact angle 38% enhancement), -12% Porosity enhancement (50 to 62%), -BSA rejection (>90%)	^43^
Ag-GO, 0.05, 0.1, 0.2, 0.5 wt%	PES, 18 wt% flat sheet UF membrane	The fabricated had relatively low protein adsorption and enhanced irreversible fouling. And showed acceptable inhibition zone against, *P. aeruginosa*, and *E. coli*.	NA	-Flux (98% enhancement), -Contact angle (17% enhancement), -Porosity (10% enhancement), -BSA rejection (>98%)	^76^
Ag-GO, 0.1, 0.5, 1 wt%	PES, 20 wt% flat sheet UF membrane	PES with 0.1wt.% of Ag-GO,	NA	-Flux (115% enhancement), -Contact angle (6% enhancement), -Dye rejection: Direct Red 16 (>98%)	^77^
Zn-rGO, 0.4 wt%, And 0.4 wt Zn NP	PVDF 18 wt% of PVDF flat sheet membrane with pure ZnO nanoparticles and ZnO-rGO nanoplates	Effect of ZnO-rGO particles decoration on stability of the mixed matrix membranes was investigated by the authors. the permeate flux of the membranes embedded with pure ZnO nanoparticles showed 7 ppb of zinc leaching for the first 3 hours of filtration, on the other hand, no Zn element was detected in the permeate flux of the membrane embedded with Zn-rGO. Providing hard evidence that decoration of metal nanoparticles on of GO prevent or delays leaching.	Investigated using ICP, no trace of nanoparticles was found after decoration on GO.	-61% of permeation flux increment, -32% BSA rejection enhancement and flux recovery ratio of 48%. -Acceptable antibacterial against *E. coli*	^78^

The agglomeration of pure silver nanoparticles was pointed out by many authors^24,68,74^ while some of the past studies did not reveal the distribution of nanoparticles in their membranes^27,67,71,72^. Various modification methods (as shown in Table 2) have been attempted to reduce the agglomeration with success^24,69–71,74^. However, based on their reports, leaching still remained a problem due to the lack of proper anchor sites for Ag nanoparticles. In this study, no agglomeration of silver nanoparticles has been observed. Hence it could be concluded that the decoration of Ag on GO help alleviating the issue of agglomeration and leaching of the nanoparticles simultaneously.

## Conclusion

This study has shown that the decoration of Ag onto GO nanoplates prevented the agglomeration of Ag especially when embedded into the membrane matrix. In addition, the GO provided the anchor sites for the Ag to attach and thus eliminated the leaching issue. The EDX mapping result presented that the silver nanoparticles distribution is even and there was no agglomeration observed. Overall, the incorporation of minimal amount of Ag-GO brought marked improvement on the membrane properties, such as flux, rejection, fouling, and hydrophilicity. Generally, Ag-GO enlarged the mean pore radius and enhanced the porosity of the polyamide membrane. Membranes embedded with Ag-GO possessed better hydrophilicity (46% enhancement) and stronger negative surface charge (−14 ± 6 to −31 ± 3.8 (mV)) as compared to pristine membrane. The combination of all these enhanced properties contributed to higher water flux (135% increment) and lower fouling propensity (40% enhancement) compared to pristine polyamide membrane. Despite the increment in flux and pore radius, the rejection of the membranes was not compromised and remained higher than 95% for both BSA and Congo red. This was due to the stronger electrostatic repulsion (more negative surface charge) and hydrophilicity of the membranes. The antimicrobial test results confirmed that the addition of Ag-GO prevented the bacterial growth on the surface of the membrane. The synergistic effect of GO static antibacterial and oligodynamic of silver nanoparticles decorated on GO restricted the growth of gram-negative *E. coli*. Overall, the incorporation of minimal amount of Ag-GO nanoplates managed to enhance the properties of the polyamide 6,6 membranes without the issues of Ag agglomeration and leaching. This shows the encouraging potential of Ag-GO as a nanofiller for the fabrication of polyamide 6,6 membranes with superior properties in water and wastewater treatment processes.

## Supplementary information

SUPPLEMENTARY INFO
