# Supplementary material for: Enhancing Morphology and Separation Performance of Polyamide 6,6 Membranes By Minimal Incorporation of Silver Decorated Graphene Oxide Nanoparticles
Source: Sci Rep. 2019 Feb 4;9:1216. doi: 10.1038/s41598-018-38060-x (PMC6362142; doi:10.1038/s41598-018-38060-x)
Supplement: Supplementary file 1 — SUPPLEMENTARY INFO [file 41598_2018_38060_MOESM1_ESM.docx]

**SUPPLEMENTARY DATA**

**ENHANCING MORPHOLOGY AND SEPARATION PERFORMANCE OF POLYAMIDE 6,6 MEMBRANES BY MINIMAL INCORPORATION OF SILVER DECORATED GRAPHENE OXIDE NANOPARTICLES**

**Ebrahim Mahmoudi^1^, Law Yong Ng^2^, Wei Lun Ang^1,3^, Ying Tao Chung^1^, Rosiah Rohani^1,3^, Abdul Wahab Mohammad*^1,3^**

*^1^Chemical Engineering Programme, Faculty of Engineering and Built Environment, Universiti Kebangsaan Malaysia, 43600 Bangi, Selangor Darul Ehsan, Malaysia.*

*^2^Department of Chemical Engineering, Lee Kong Chian Faculty of Engineering and Science, Universiti Tunku Abdul Rahman, Jalan Sungai Long, Bandar Sungai Long, Cheras, 43000 Kajang, Selangor Darul Ehsan, Malaysia.*

*^3^Centre for Sustainable Process Technology (CESPRO), Faculty of Engineering and Built Environment, Universiti Kebangsaan Malaysia, 43600 Bangi, Selangor Darul Ehsan, Malaysia.*

b

a


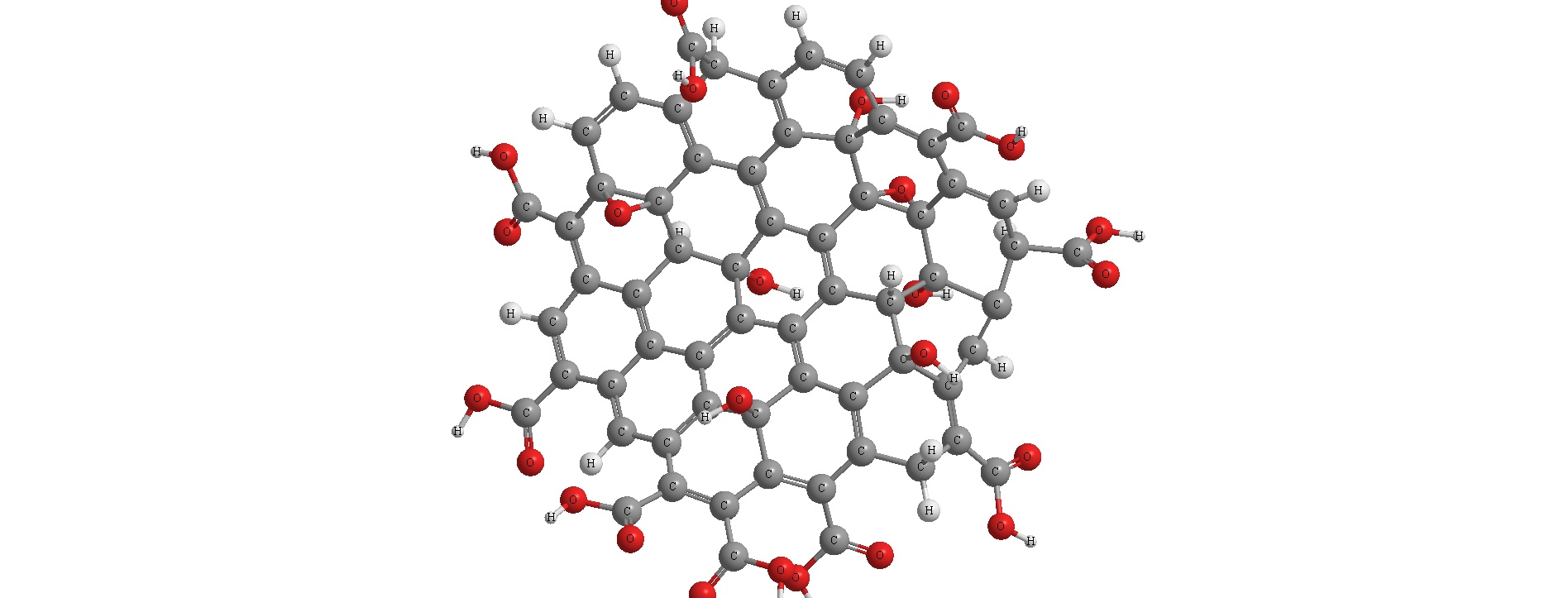

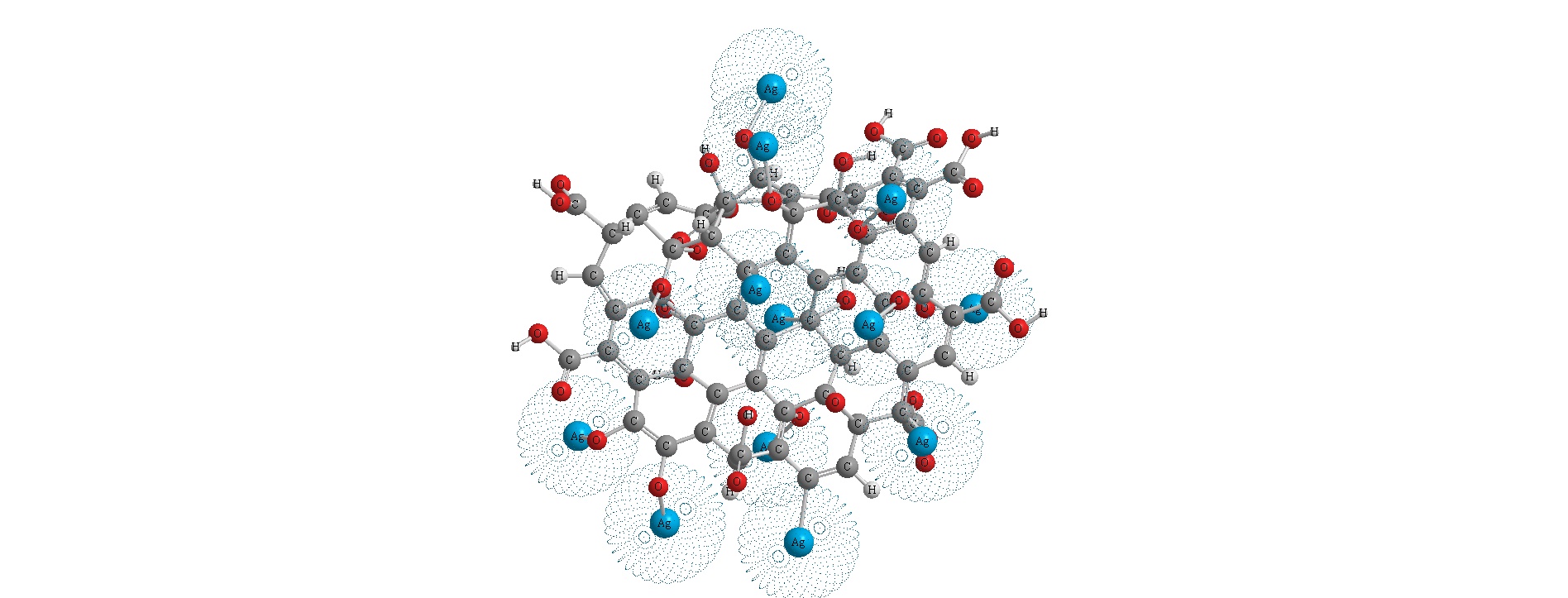


Figure S1 a: Schematic of graphene oxide, b: Schematic of silver decorated graphene oxide


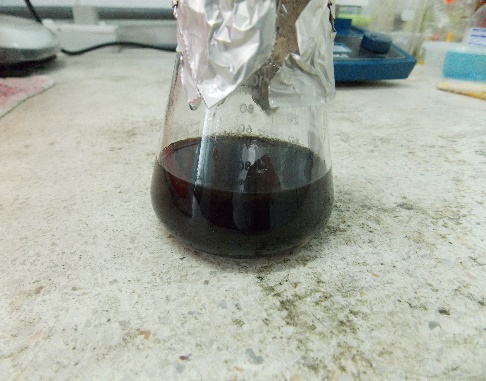

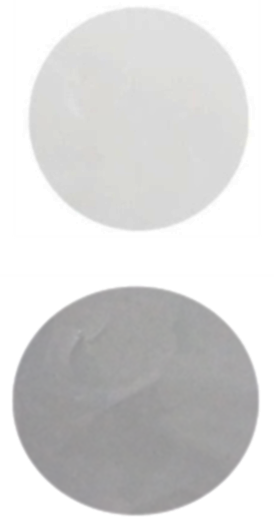


a

c

b

d


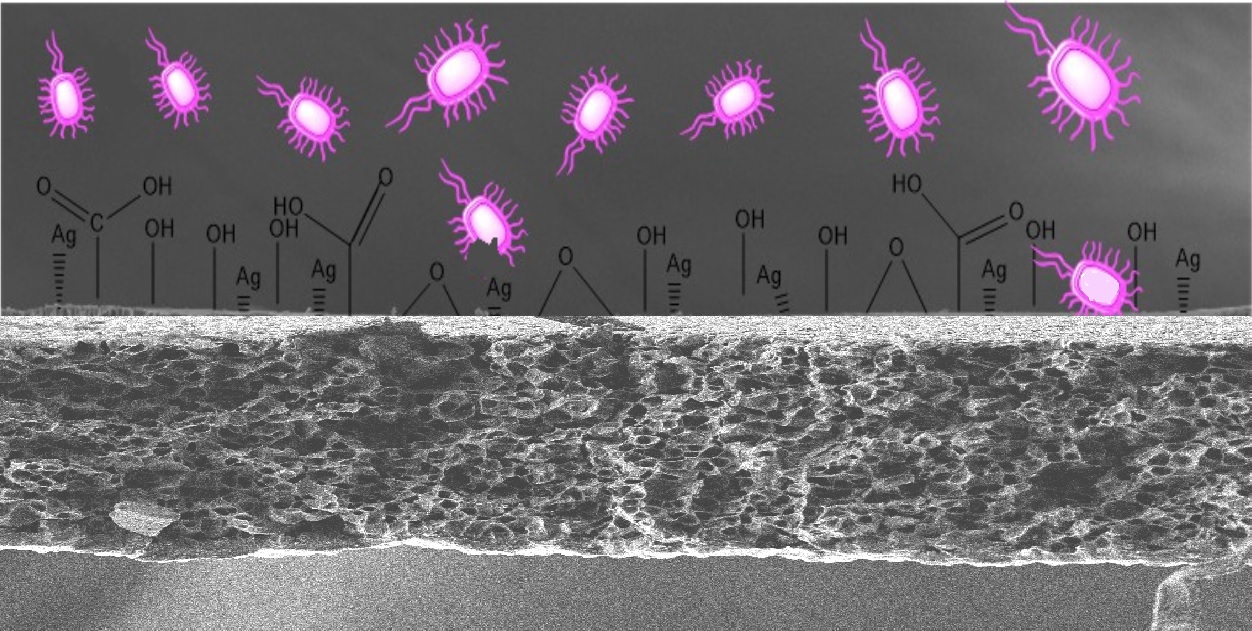


**
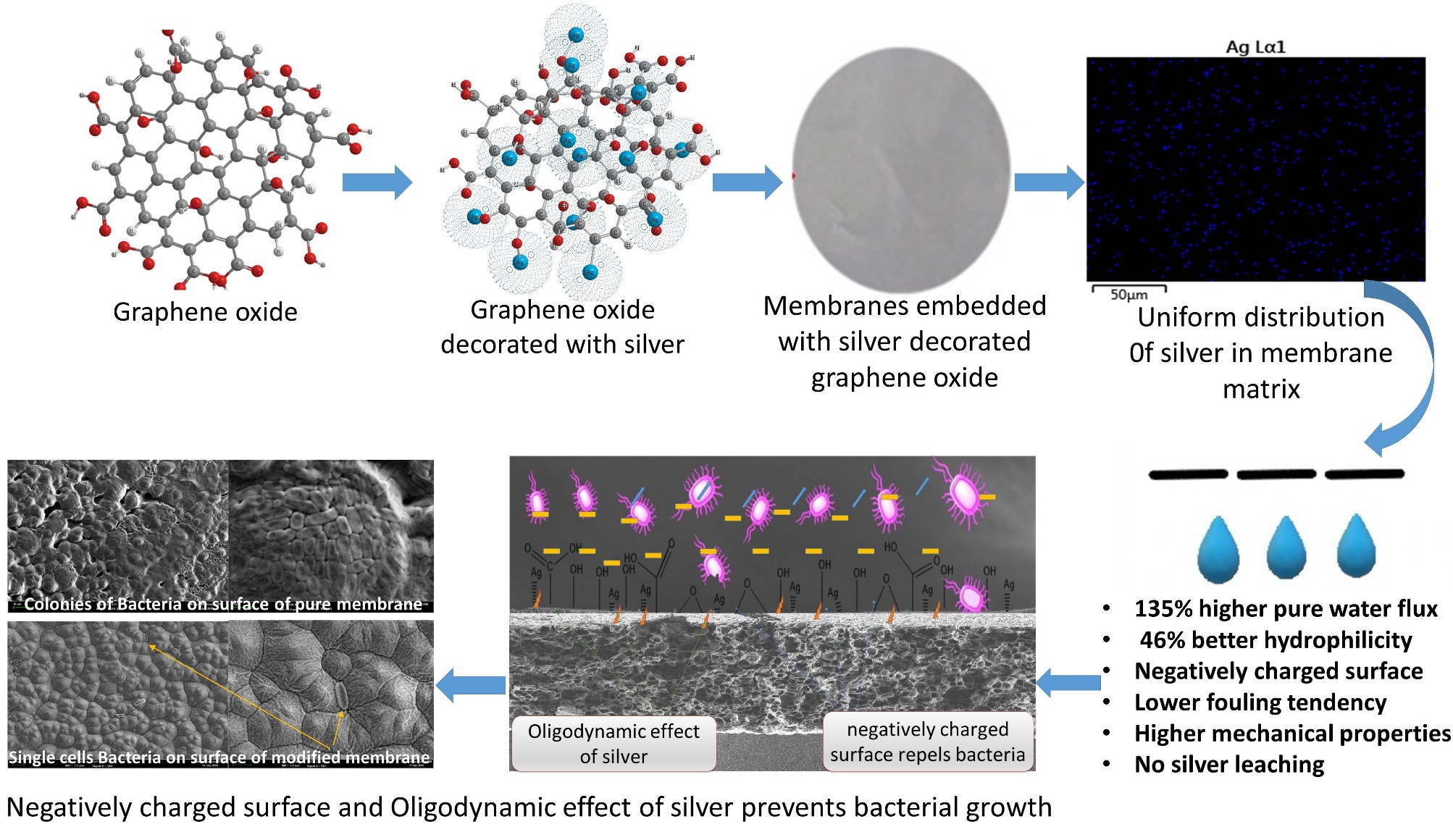
**Figure S2 Images of a: casting solution after addition of silver decorated graphene oxide, b: pure membrane(NY1), c: membrane with 0.8% of silver decorated graphene oxide (NY4), d: the schematic of the membrane surface, e: The schematic of the process

e

**Table S1 Assignment of FTIR spectra of polyamide 6,6 and Ag-GO/polyamide 6,6 membranes**

| No. | Assignment | FTIR Frequency (cm^-1^) of polyamide 6,6 (NY1) | FTIR Frequency (cm^-1^) of Ag-GO/polyamide 6,6 (NY4) |
| --- | --- | --- | --- |
| 1 | N-H stretching | 3299 | 3292 |
| 2 | C-H asymmetric stretching | 2934 | 2923 |
| 3 | CH2 asymmetric stretching | 2859 | 2853 |
| 4 | Amide I stretching | 1633 | 1630 |
| 5 | Amide II stretching/ CH_2_ | 1538 | 1533 |
| 6 | N-H deformation/ CH_2_ | 1474 | 1463 |
| 7 | N-H deformation/ CH_2_ | 1372 | 1368 |
| 8 | C-CH symmetric bending | 1277 | 1273 |
| 9 | C-CH symmetric bending | 1199 | 1197 |
| 10 | C-CH stretching | 935 | 934 |
| 11 | C-CH bending | 688 | 682 |
| 12 | O=C-N bending | 580 | 577 |


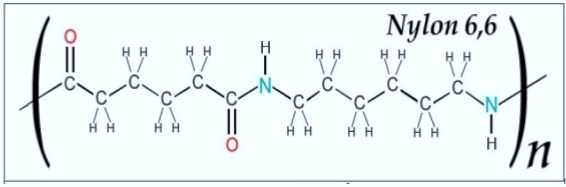


Figure S3. Nylon 6,6 structure.


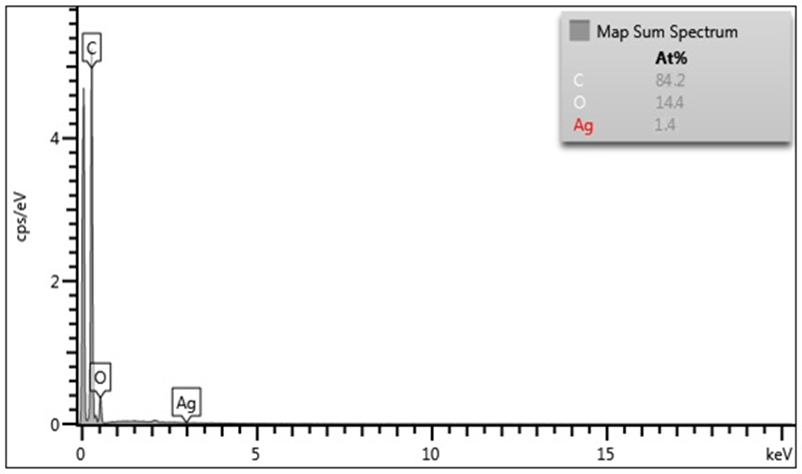

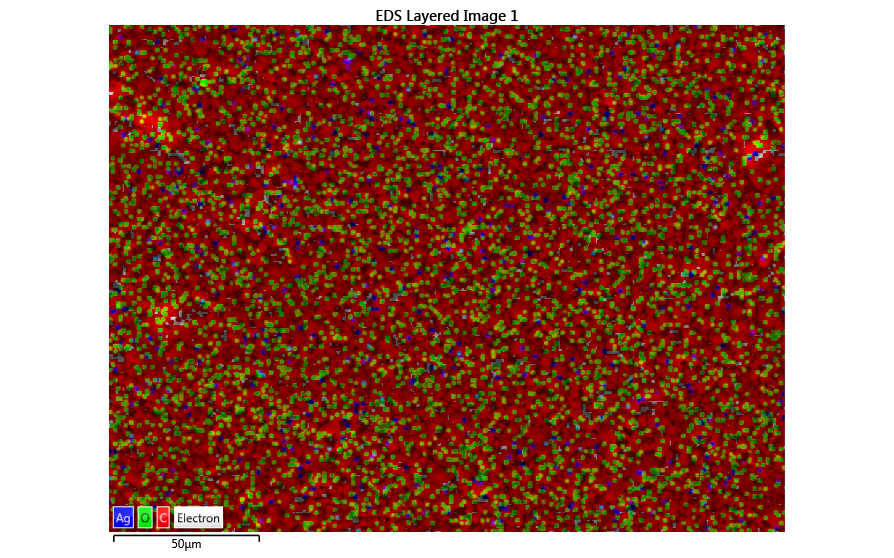

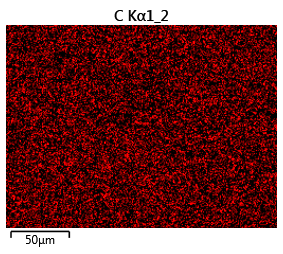

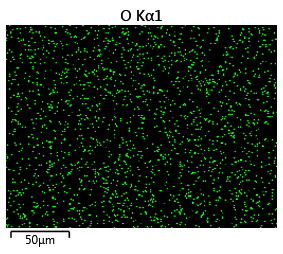

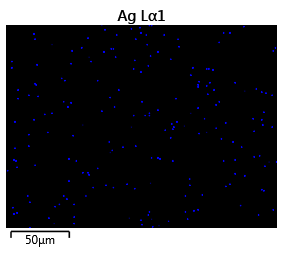


e

d

c

b

a

Figure s.4 a: EDX and FESEM mapping images of NY2 membrane c: Carbon, d: Oxygen and e: Silver mapping.

**



**

b

a

Figure S.5 SEM images of the prepared membranes. (4KX) A:pure nylon membrane(NY1), d :membrane(NY4).

Table S2 ICP analysis results for Permeate of the Membranes

| **Sample** | **The concentration of Ag (ppm)** | **Remarks** |
| --- | --- | --- |
| NY2 Permeate | 0.00 | No leaching of Ag from the membrane |
| NY3 Permeate | 0.00 | No leaching of Ag from the membrane |
| NY4 Permeate | 0.00 | No leaching of Ag from the membrane |
| NY5 Permeate | 0.00 | No leaching of Ag from the membrane |


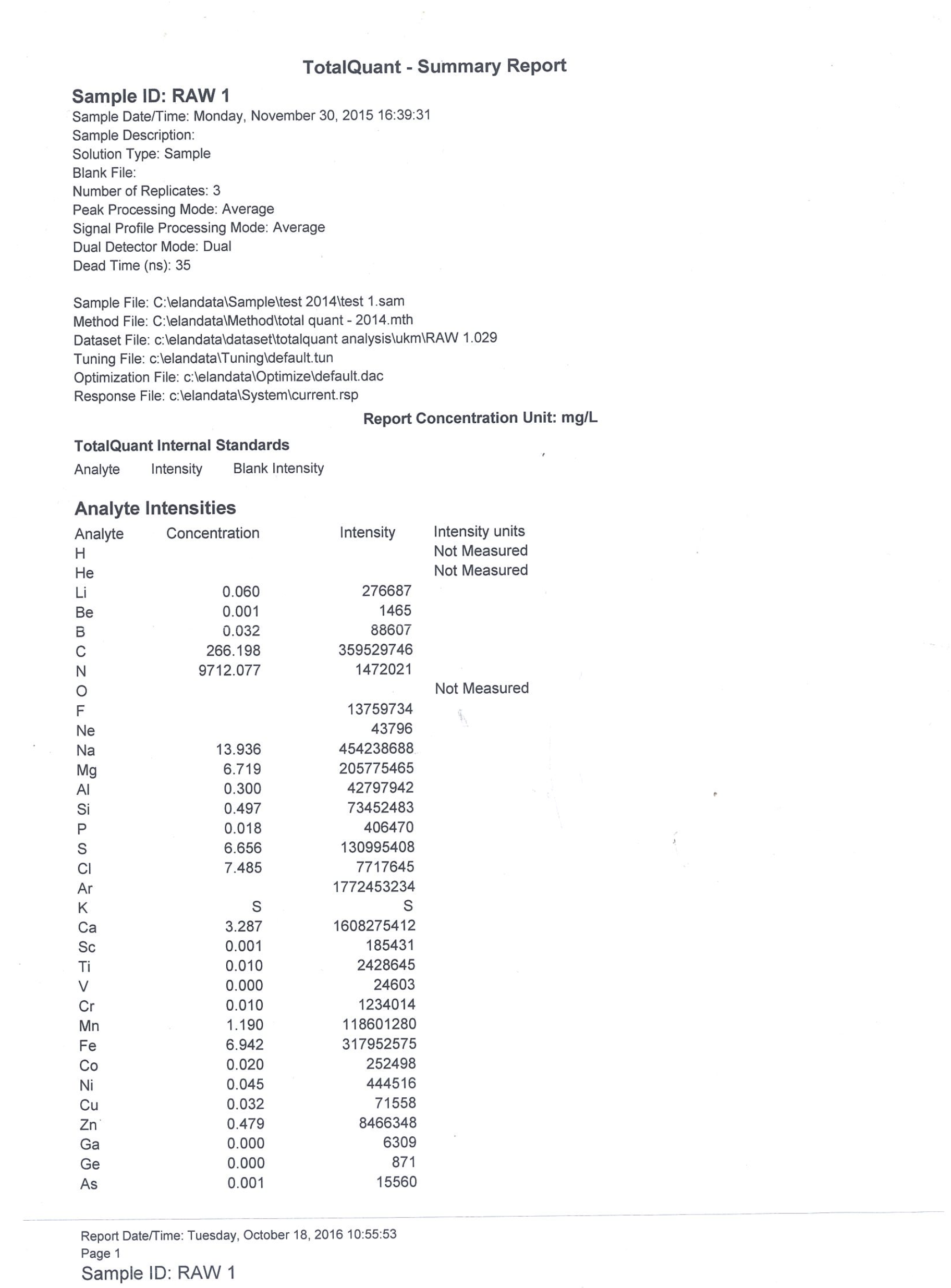

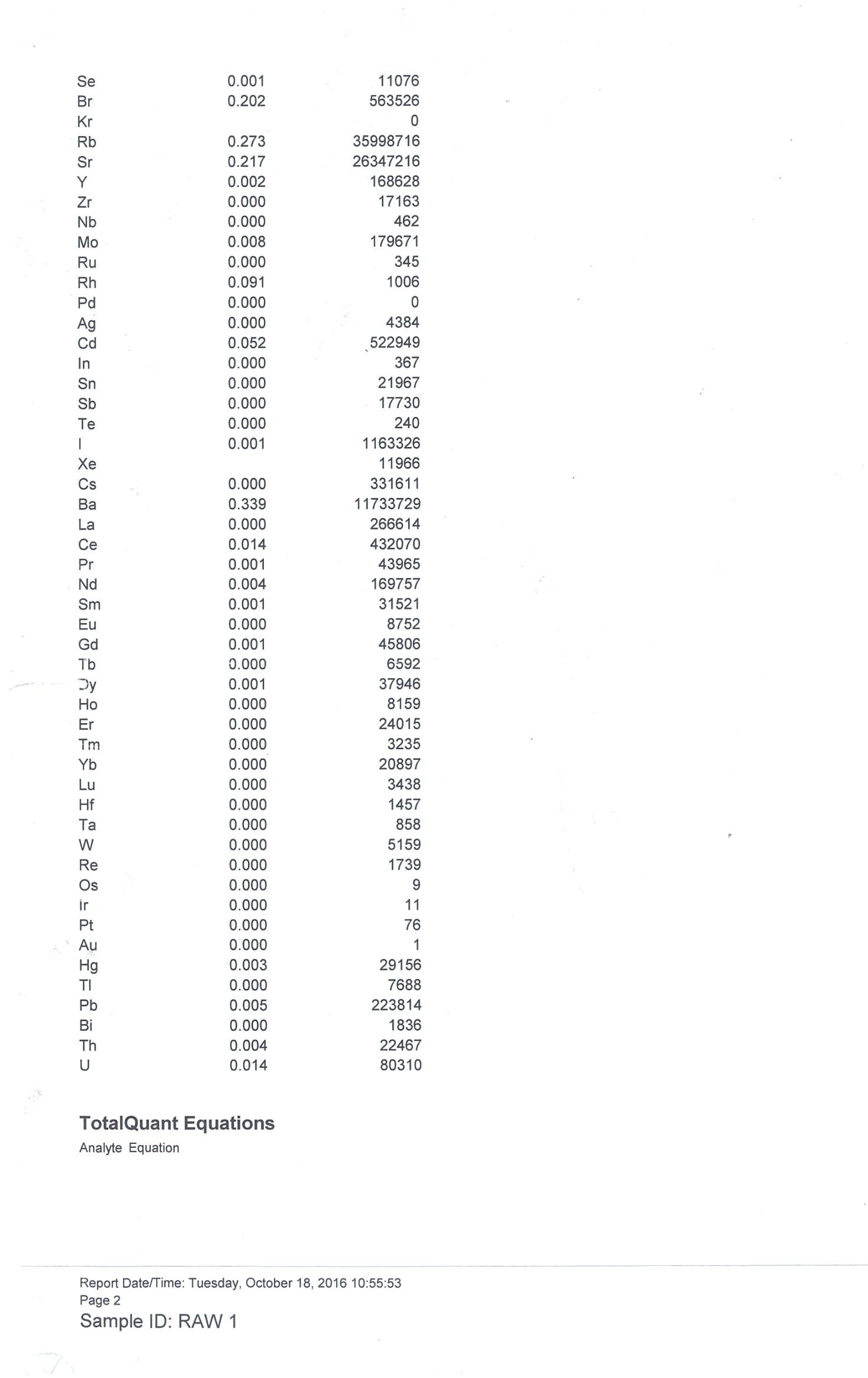

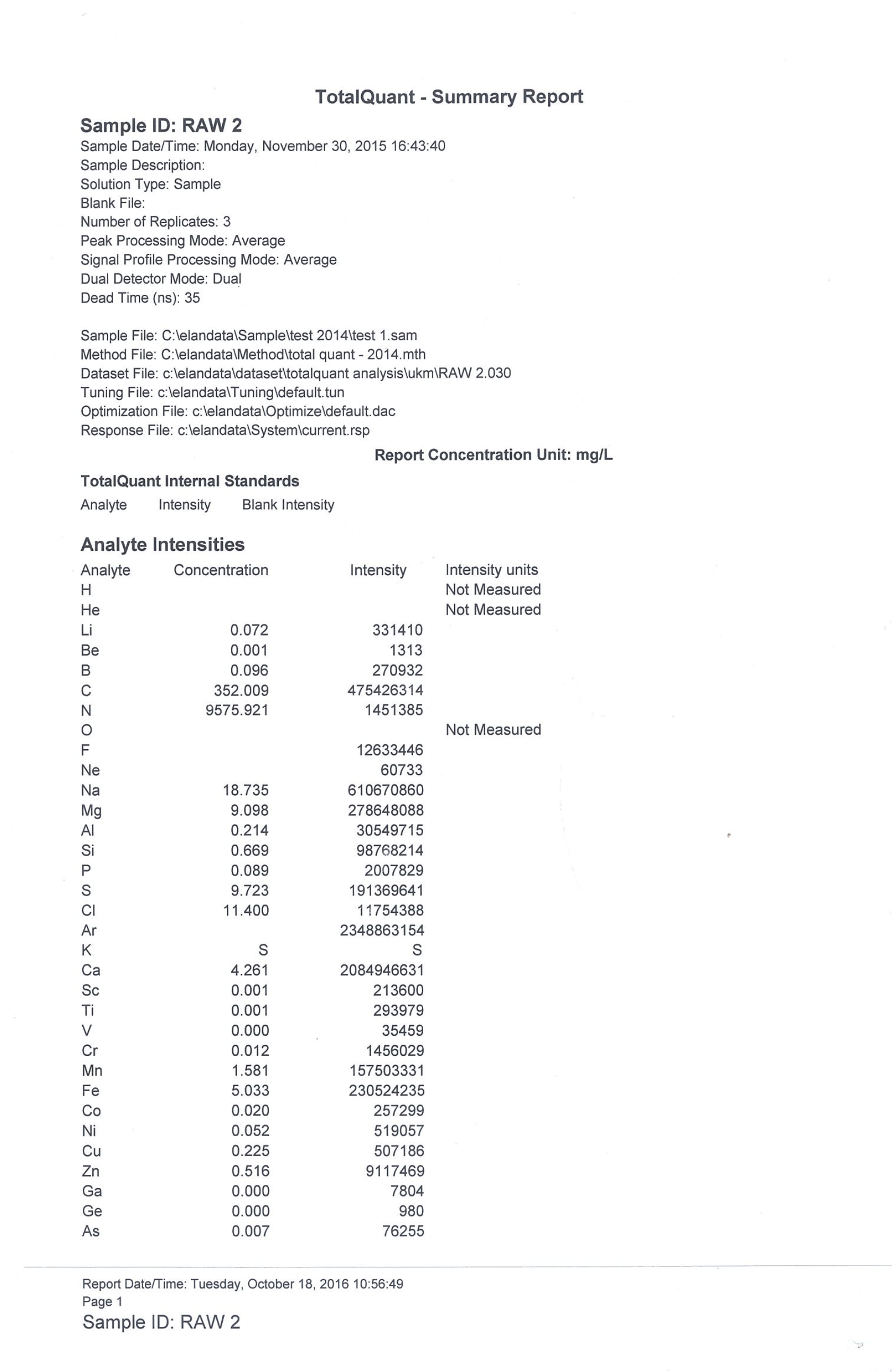

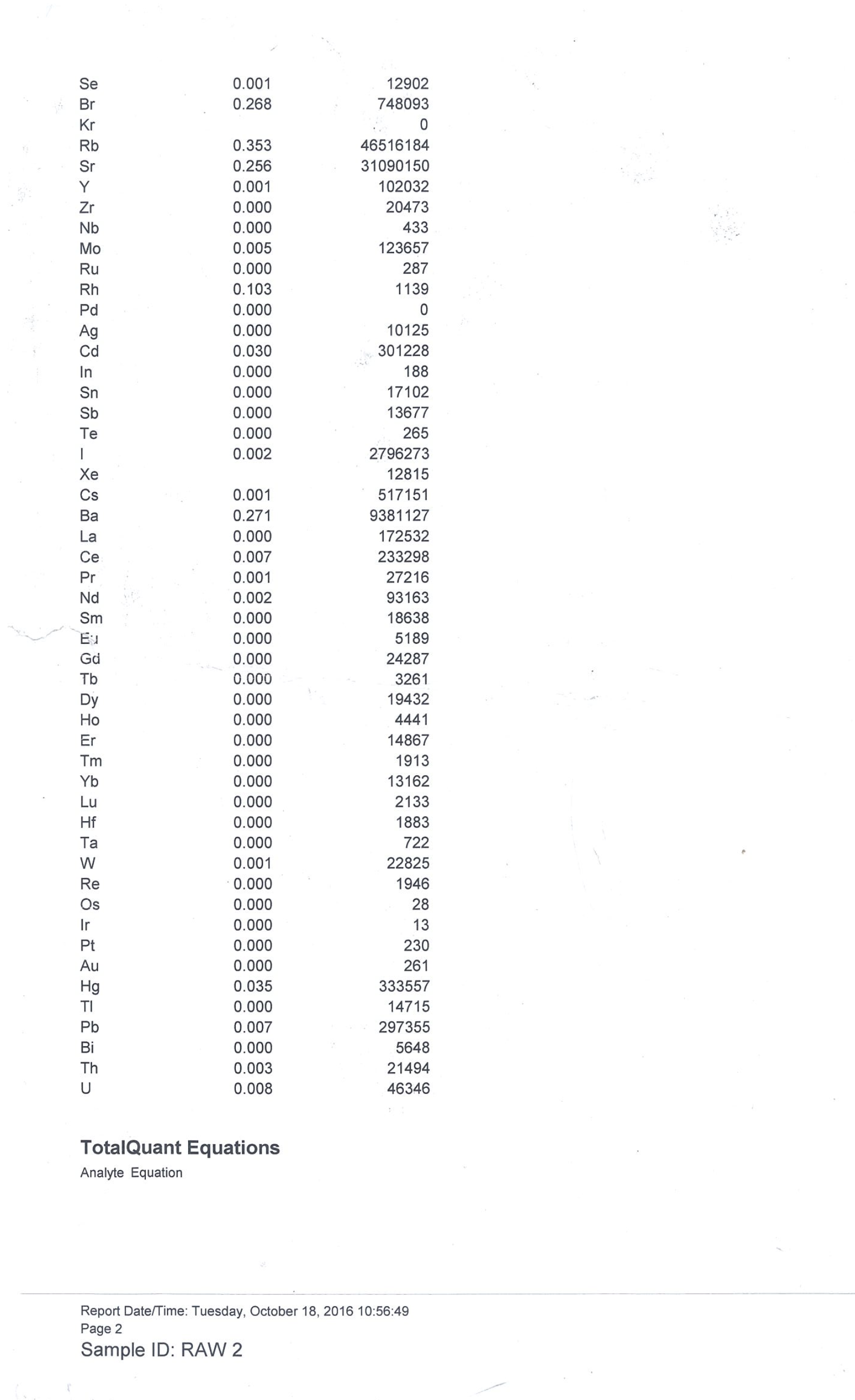


Figure s6 ICP-MS results of the membranes permeate flux ( NY2(Raw1) and NY5(raw2) ) shows no sign of silver ions
